# The polymeric immunoglobulin receptor-like protein from *Marsupenaeus japonicus* is a receptor for white spot syndrome virus infection

**DOI:** 10.1371/journal.ppat.1007558

**Published:** 2019-02-06

**Authors:** Guo-Juan Niu, Shuai Wang, Ji-Dong Xu, Ming-Chong Yang, Jie-Jie Sun, Zhong-Hua He, Xiao-Fan Zhao, Jin-Xing Wang

**Affiliations:** 1 Shandong Provincial Key Laboratory of Animal Cells and Developmental Biology, School of Life Sciences, Shandong University, Qingdao, Shandong, China; 2 State Key Laboratory of Microbial Technology, Shandong University, Qingdao, Shandong, China; 3 Institutes of Biology and Medical Sciences, Soochow University, Suzhou, Jiangsu, China; The Ohio State University, UNITED STATES

## Abstract

Viral entry into the host cell is the first step towards successful infection. Viral entry starts with virion attachment, and binding to receptors. Receptor binding viruses either directly release their genome into the cell, or enter cells through endocytosis. For DNA viruses and a few RNA viruses, the endocytosed viruses will transport from cytoplasm into the nucleus followed by gene expression. Receptors on the cell membrane play a crucial role in viral infection. Although several attachment factors, or candidate receptors, for the infection of white spot syndrome virus (WSSV) were identified in shrimp, the authentic entry receptors for WSSV infection and the intracellular signaling triggering by interaction of WSSV with receptors remain unclear. In the present study, a receptor for WSSV infection in kuruma shrimp, *Marsupenaeus japonicus*, was identified. It is a member of the immunoglobulin superfamily (IgSF) with a transmembrane region, and is similar to the vertebrate polymeric immunoglobulin receptor (pIgR); therefore, it was designated as a pIgR-like protein (*Mj*pIgR for short). *Mj*pIgR was detected in all tissues tested, and its expression was significantly induced by WSSV infection at the mRNA and protein levels. Knockdown of *MjpIgR*, and blocking *Mj*pIgR with its antibody inhibited WSSV infection in shrimp and overexpression of *Mj*pIgR facilitated the invasion of WSSV. Further analyses indicated that *Mj*pIgR could independently render non-permissive cells susceptible to WSSV infection. The extracellular domain of *Mj*pIgR interacts with envelope protein VP24 of WSSV and the intracellular domain interacts with calmodulin (*Mj*CaM). *Mj*pIgR was oligomerized and internalized following WSSV infection and the internalization was associated with endocytosis of WSSV. The viral internalization facilitating ability of *Mj*pIgR could be blocked using chlorpromazine, an inhibitor of clathrin dependent endocytosis. Knockdown of *Mjclathrin* and its adaptor protein *AP-2* also inhibited WSSV internalization. All the results indicated that *Mj*pIgR-mediated WSSV endocytosis was clathrin dependent. The results suggested that *Mj*pIgR is a WSSV receptor, and that WSSV enters shrimp cells via the pIgR-CaM-Clathrin endocytosis pathway.

## Author summary

White Spot Syndrome Virus (WSSV) is one of the most virulent pathogens in shrimp farming. Several viral candidate receptors, or attachment factors were reported in previous studies, however, most of them are not authentic transmembrane proteins. In particular, the protein receptor(s) required the intracellular signaling triggering by interaction of WSSV with receptors remain unclear. In the present study, a polymeric immunoglobulin receptor (pIgR) like protein, a bona fide transmembrane receptor, was identified in kuruma shrimp, *Marsupenaeus japonicus* (*Mj*pIgR for short). Knockdown of *Mj*pIgR by RNA interference, and blocking it by its antibody prevented WSSV infection in shrimp and overexpression of *Mj*pIgR facilitated the invasion of WSSV. Further study found that *Mj*pIgR could independently render non-permissive cells susceptible to WSSV infection. The extracellular cellular domain of *Mj*pIgR interacts with envelope protein VP24 of WSSV and the intracellular domain interacts with calmodulin (*Mj*CaM). *Mj*pIgR was oligomerized and internalized following WSSV infection and the internalization was associated with endocytosis of WSSV. The viral internalization facilitating ability of *Mj*pIgR could be blocked using chlorpromazine, an inhibitor of clathrin dependent endocytosis, indicating that *Mj*pIgR-mediated WSSV endocytosis was clathrin dependent. The results suggested that *Mj*pIgR is a WSSV receptor, and that WSSV enters shrimp cells via the pIgR-CaM-Clathrin endocytosis pathway. This study provides a new target for WSSV control in shrimp aquaculture.

## Introduction

Viral infection process is a very complex interaction and consists of multiple steps [1]. It starts with virion attachment to the host cell membrane, followed by specific binding to receptors. Viral receptor engagement allows viruses either to release their genome into the cell directly at the plasma membrane, or to enter cells through endocytosis. Endocytosis is highly complex and dynamic, and involves recycling, trafficking, maturation and fusion of endocytic vesicles [2]. For DNA viruses and a few RNA viruses, the endocytosed viruses will traffic from cytoplasm into the nucleus for gene expression [1,3]. To enter the cytoplasm of host cells, viruses can adopt two main strategies, receptor-mediated endocytosis and endocytosis-independent receptor-mediated entry [4]. Viruses can use specific cell membrane receptors to enter and infect host cells, which determines the host specificity, tissue tropism and cell type a virus can infect [5,6]. Several classes of molecules are utilized as receptors by different viruses, such as sialic acid moieties, integrins, and some immunoglobulin-like superfamily (IgSF) proteins in vertebrates [7]. Some viruses use various types of receptors to attach to and enter into cells. For example, the receptors for hepatitis C virus (HCV) infection include heparin sulfate [8], low-density lipoprotein receptor [9], transferrin receptor 1 [10], B type scavenger receptor [11] and occludin [12] in mammals.

Cell-adhesion molecules can be divided into four protein families: Integrins, selectins, IgSF, and cadherins [13]. They are usually expressed on the cell surface and have diverse functions. Among them, the IgSF is a large protein superfamily of cell surface and soluble proteins that are involved in recognition, binding, adhesion, and immunity [14]. IgSF members have diverged in sequence and function; however, the definitive characteristic of the members is the presence of one or more immunoglobulin (Ig)-like domains [15]. The polymeric immunoglobulin receptor identified in vertebrates is a member of the IgSF. As a type I transmembrane glycoprotein, polymeric immunoglobulin receptor (pIgR) is widely expressed in epithelial cells [16]. The pIgR protein in different species shares four similar components: An intracellular region, a transmembrane region, a cleavage region, and an extracellular ligand-binding region (secretory component, SC) [17]. The Ig domains are located in the extracellular region; therefore, the N-terminal ligand-binding domain plays central roles in binding polymeric immunoglobulins (pIg). PIgR acts as the receptor for pIg and transports pIgA/pIgM across intestinal epithelial cells (IECs) in vertebrates [18,19]. In addition, pIgR and SC-mediated protection prevent the invasion of pathogenic microorganism at mucosal surfaces [20,21]. Interestingly, some studies on pIgR have found that certain microorganisms, such as *Streptococcus pneumoniae*, hijack pIgR to their own benefit during the invasion of host cells [22,23,24].

Diverse groups of viruses bind to IgSF proteins at the cell surface to mediate cell entry [7]. For example, the cell surface CD4 glycoprotein carries four functional domains, and three of them resemble Ig variable regions, CD4 was confirmed as an important receptor of Human Immunodeficiency Virus (HIV) [25]. It is now known that entry of HIV-1 into lymphoid cells requires the cooperation of three host-cell proteins, the primary receptor CD4, a chemokine co-receptor (CCR5 or CXCR4) and an oxidoreductase protein disulfide isomerase (PDI) and the viral envelope glycoproteins gp120 and gp41 [26,27,28,29]. Viral gp120 attaches the virus to the cell by binding to host CD4. It was found that CD4 also has a binding site for PDI and forms a PDI-CD4-gp120 complex [27]. Another example is where the Adeno-associated virus receptor (AAVR) serves as an important receptor for the invasion of Adeno-associated virus (AAV). AAVR with five Ig-like domains, also known as polycystic kidney disease (PKD) domains, was captured by AAV during breaking of the defensive system of different cell lines [30].

White Spot Syndrome Virus (WSSV) is one of the most virulent pathogens in shrimp farming [31]. Studies on viral candidate receptors involved in WSSV infection can provide useful information for viral disease control. There were several reports about WSSV attachment proteins or candidate receptors in shrimp, such as *Penaeus monodon* Rab7 binding to WSSV envelope protein VP28, which is beneficial for WSSV infection [32], and a chitin-binding protein (CBP) in *P*. *monodon* interacts with 11 WSSV envelope proteins, which can reduce and delay mortality upon WSSV challenge in the neutralization assay [33,34]. Beta-integrin interacts with VP187, which can mediate WSSV infection [35]. Glucose transporter 1 interacts with VP53A, which is related with entry of WSSV into host cells [36]. Laminin binding to VP31 mediates WSSV infection [37] and a soluble C-type lectin (*Mj*svCL) interacts with VP28 and calreticulin, which facilitates WSSV infection in shrimp [38]. Other studies found that some proteins interact with WSSV proteins to resist WSSV infection. F_1_-ATP synthase beta subunit of *Litopenaeus vannamei* binds to WSSV and attenuates WSSV infection [39]. Scavenger receptor C of *Marsupenaeus japonicus* interacts with VP19 of WSSV and β-arrestin mediates clathrin dependent endocytosis of WSSV, which can restrict virus proliferation [40]. These reports advanced our understanding of WSSV entry receptors.

Viral receptors play important roles in the initial step of viral infection, and are ideal targets for antiviral intervention. Usually, interactions of virus with the receptors can elicit two types of signaling, viral particle conformational changes, and intracellular signals triggering specific cellular responses. In many cases, virus can usurp the signaling systems of host cells to create a favorable environment for their own amplification [41]. Among the reported WSSV candidate receptors that are beneficial for WSSV infection, only the β-integrin is an authentic transmembrane protein; therefore, further study of WSSV entry receptors is required. On the other hand, the signaling induced by WSSV interactions with receptors remains unknown. In the present study, we identified an IgSF cell adhesion molecule that was similar to poly immunoglobin receptor (pIgR) of vertebrates from *Marsupenaeus japonicus*, and designed it as *Marsupenaeus japonicus* pIgR like protein (*Mj*pIgR). *Mj*pIgR is a type I transmembrane protein, and was significantly upregulated in shrimp challenged with WSSV. Knockdown of *Mj*pIgR in shrimp decreased the numbers of WSSV. Meanwhile, overexpression of *Mj*pIgR increased WSSV infection. The intracellular signaling triggering by interaction of WSSV with *Mj*pIgR was investigated. *Mj*pIgR interacted with *Mj*CaM and the viral internalization was clathrin dependent. Our studies revealed that pIgR is a receptor for the invasion of WSSV into shrimp.

## Results

### *Mj*pIgR was upregulated in shrimp challenged by WSSV

In our transcriptome sequence analysis, we found a pIgR-like molecule that was upregulated by 4 to 6 folds in shrimp challenged with WSSV. Therefore, we chose this molecule for further study. The full-length *MjpIgR* cDNA is 1686 bp and encodes a protein of 562 amino acid residues (GenBank Accession no. MH051890). *Mj*pIgR contains a signal peptide; an extracellular domain, including an IG domain and two IG-like domains; a transmembrane region; and an intracellular region (S1 and S2 Figs). *Mj*pIgR is clustered with the vertebrate pIgR group (S3 Fig).

**Fig 1:**
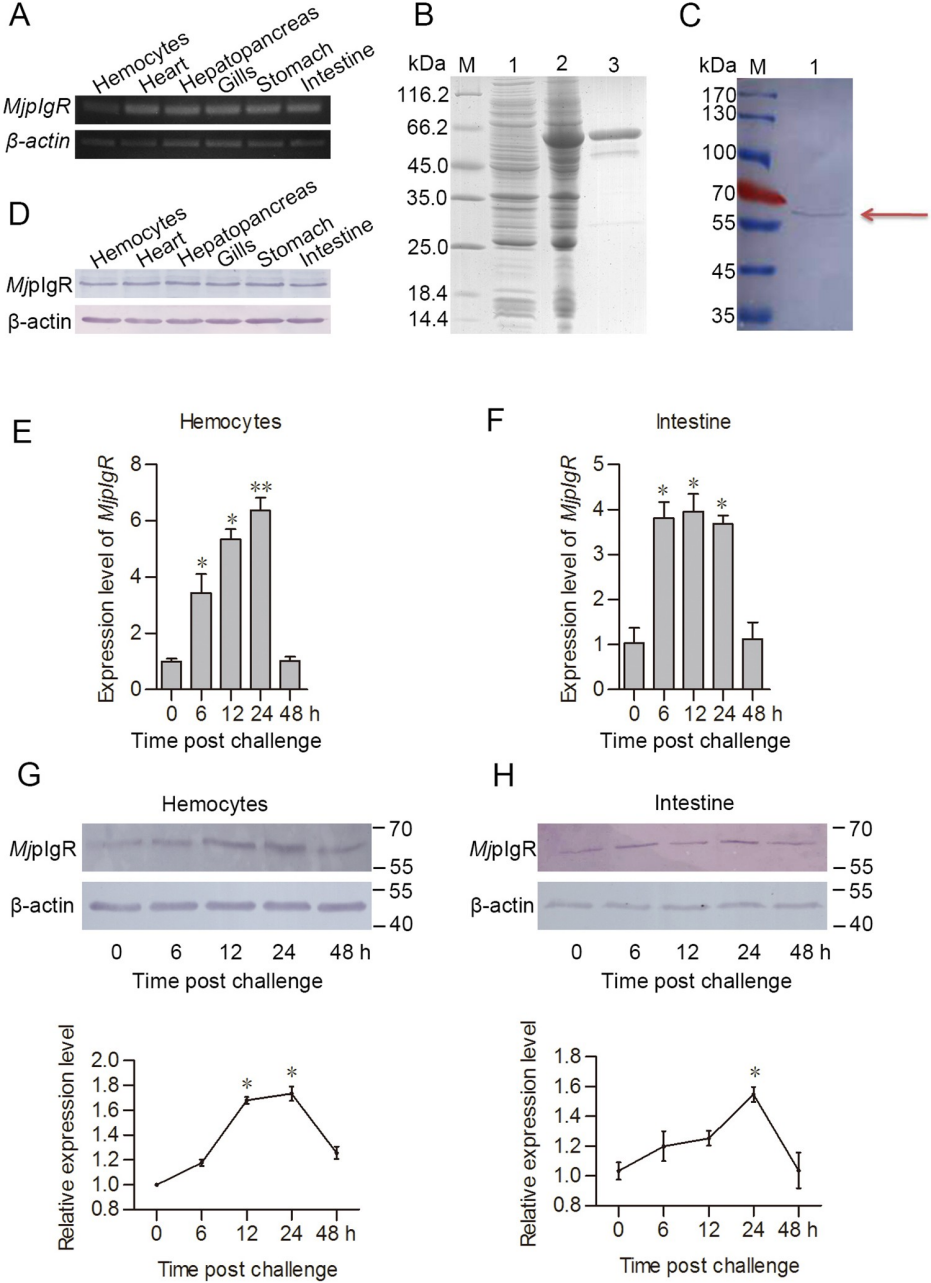
*Mj*pIgR was upregulated in shrimp after WSSV challenge. **A**, The tissue distribution of *MjpIgR* in shrimp at the mRNA level. **B**, Recombinant expression and purification of the extracellular region of *Mj*pIgR in *E*. *coli*. Lane 1, total proteins from *E*. *coli* with *Mj*pIgR-pGEX4T-1, without IPTG induction; lane 2, total proteins from the *E*. *coli* with IPTG induction; lane 3, purified recombinant *Mj*pIgR; lane M, protein molecular mass marker. **C**, *Mj*pIgR in normal hemocytes of shrimp was detected using western blotting with *Mj*pIgR polyclonal antibodies. Lane M, protein marker; lane 1, *Mj*pIgR in hemocytes detected using the *Mj*pIgR polyclonal antibodies. **D**, The tissue distribution of *Mj*pIgR in different tissues was investigated using western blotting. **E**-**F**, Expression patterns of *MjpIgR* in hemocytes (**E**) and intestine (**F)**, as detected using qPCR. The data were analyzed statistically using Student’s *t* test. **G-H**, *Mj*pIgR expression patterns at the protein level in hemocytes (**G)** and intestine (**H)** of shrimp after WSSV challenge, as analyzed using western blotting. The lower panels of (**G)** and (**H)** show statistical analysis for three replicates. The results are expressed as the mean ± SD. *, *p*< 0.05; **, *p* < 0.01.

*MjpIgR* mRNA is expressed in hemocytes and in all other tested organs including heart, hepatopancreas, gills, stomach, and intestine analyzed by RT-PCR (Fig 1A). The specificity of the *MjpIgR* ORF primers was confirmed by using other samples from *Litopenaeus vannamei* and *Procambarus clakii*, which shows no any band by PCR amplification (S4 Fig). The extracellular SC of *Mj*pIgR protein was recombinantly expressed in *E*. *coli* (Fig 1B) and anti-*Mj*pIgR polyclonal antibodies were prepared (Fig 1C). The *Mj*pIgR protein was also widely distributed in hemocytes and other organs, as revealed via western blotting analysis (Fig 1D). All the results indicated that *Mj*pIgR is ubiquitously expressed in the shrimp.

We performed a time course expression analysis of *Mj*pIgR transcription and translation in hemocytes and intestine. The qPCR results showed that *MjpIgR* transcription was upregulated from 6 to 24 h in hemocytes and intestine of shrimp after WSSV challenge (Fig 1E and 1F). The *Mj*pIgR protein level was also upregulated similarly to mRNA level (Fig 1G and 1H). These results suggested that *Mj*pIgR is involved in WSSV infection and its increased expression prompted us to explore the detailed functions of *Mj*pIgR in shrimp immunity.

### *Mj*pIgR promoted the infection of WSSV

**Fig 2:**
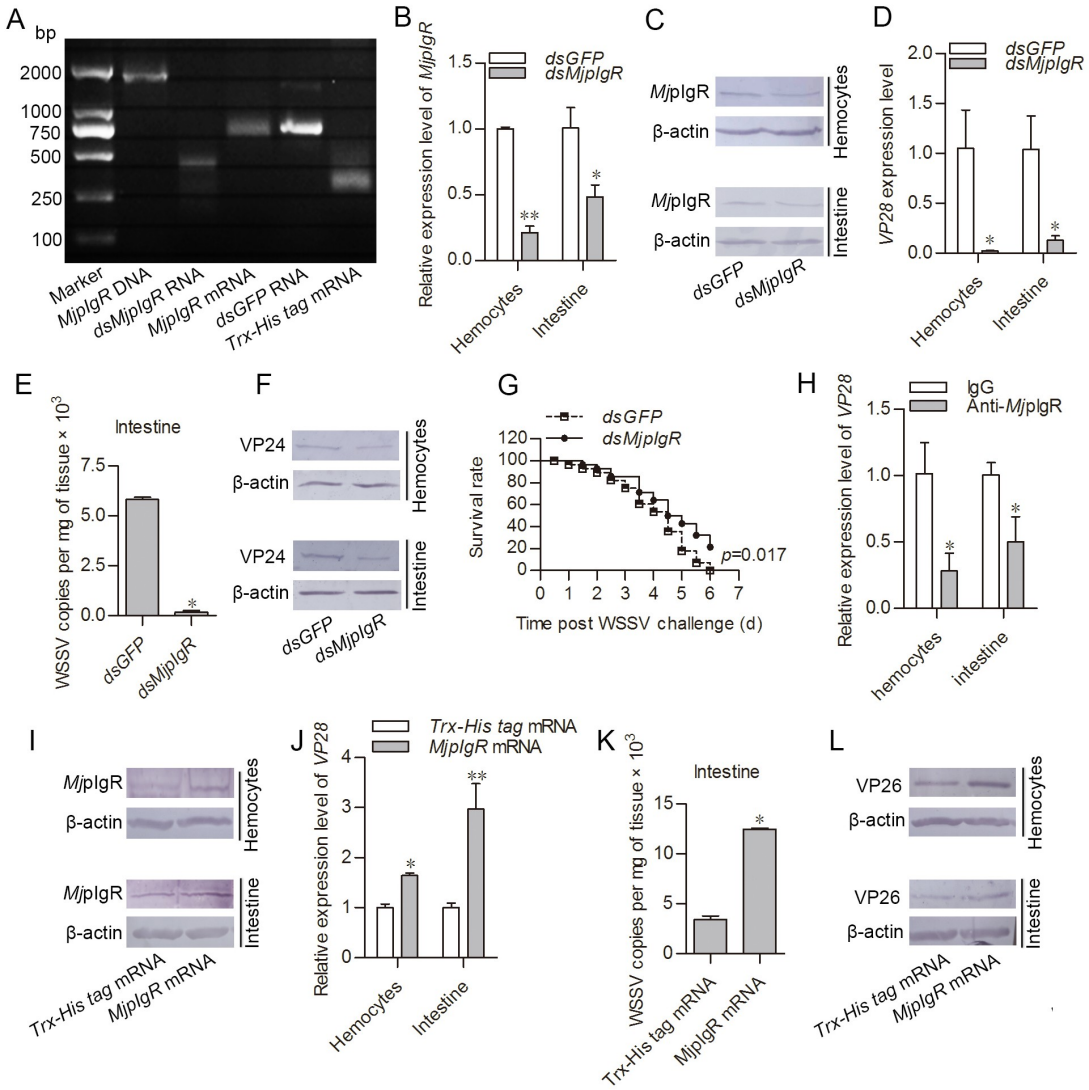
*Mj*pIgR promotes WSSV proliferation in shrimp. **A**, Agarose gel electrophoresis of *MjpIgR* cDNA, *dsMjpIgR* RNA, *MjpIgR* mRNA, and control groups (*dsGFP* RNA and *Trx-His tag* mRNA). **B-C**, Efficiency of *MjpIgR*-RNAi in hemocytes and intestine, as determined using qPCR **(B)** and western blotting analysis (**C**). **D**, The expression of WSSV *VP28* in *MjpIgR* knockdown shrimp infected with WSSV was detected using qPCR. **E**, The number of copies of WSSV in *MjpIgR*-knockdown shrimp and *dsGFP*-injection shrimp was analyzed using qPCR on Genomic DNA from the intestine. **F**, The translation level of WSSV envelope protein VP24 was detected using western blotting. **G**, The survival rate of shrimp. Shrimp were divided into *dsGFP* and *dsMjpIgR* groups. After RNAi for 24 h, the two groups were infected with WSSV for an additional 24 h. Dead shrimp were then monitored every half-day and the survival rate was calculated as the live shrimp/total shrimp ratio. Significant differences between the two groups are marked with the *P* value. Significant differences were analyzed statistically using the GraphPad Prism 5.0 software. **H**, Purified rabbit pre-serum and purified anti-*Mj*pIgR antibodies were injected into shrimp and then WSSV infection was performed. The *vp28* expression levels were detected in hemocytes and intestine using qPCR. **I**, The efficiency of *Mj*pIgR overexpression levels in hemocytes and intestine, as determined by western blotting. **J**, *Vp28* expression was detected in shrimp after *Mj*pIgR overexpression using qPCR. **K**, WSSV copies in the intestine were analyzed using qPCR. **L**, WSSV envelope protein VP26 was determined by western blotting. *, *p* < 0.05; **, *p* < 0.01.

To explore the *Mj*pIgR functions in WSSV infection, RNA interference, antibody blocking assays, and mRNA overexpression of *Mj*pIgR were performed. WSSV proliferation in shrimp was analyzed via qPCR (by testing vp28 expression level and WSSV copies) and western blotting (using VP24 or VP26 as indicators). The dsRNA and mRNA of *Mj*pIgR were generated (Fig 2A). Twenty-four hours after the injection of *dsMjpIgR* in shrimp, *Mj*pIgR was observed to be knocked down at the mRNA and protein levels (Fig 2B and 2C). The shrimp was then injected with WSSV. The WSSV levels in hemocytes and intestine of the *dsMjpIgR*-injection group were significantly reduced compared with those injected with *dsGFP* at 24 h post injection (Fig 2D). Meanwhile, the number of copies of WSSV decreased significantly in the intestine of *MjpIgR*-knockdown shrimp (Fig 2E). The WSSV protein level detected by anti-VP24 antibodies also decreased in hemocytes and intestine in the *dsMjpIgR* injection group compared with that in the control group (Fig 2F). The survival rate of shrimp was also analyzed after RNAi of *Mj*pIgR in shrimp followed WSSV injection. The results showed that the *dsMjpIgR* injection group had a much higher survival rate compared with that of the *dsGFP* group (Fig 2G). In addition, the antibody blocking assay showed *vp28* expression was decreased in the anti-*Mj*pIgR injected shrimp, and suggested that the anti-*Mj*pIgR antibodies blocked the binding site on the membrane in hemocytes and intestine (Fig 2H). Taken together, these results suggested that *Mj*pIgR promoted WSSV proliferation in shrimp.

To further explore its function, overexpression of *Mj*pIgR was performed by *Mj*pIgR mRNA injection using Trx-His tag mRNA as a control. The *Mj*pIgR protein was successfully expressed in hemocytes and intestine of the *Mj*pIgR-overexpression group at 24 h post-mRNA injection (Fig 2I). The shrimp were then challenged with WSSV. WSSV proliferation in the *Mj*pIgR-overexpression group increased dramatically compared with that in the Trx-His tag mRNA groups (Fig 2J). Similar results were obtained in the intestine by testing the number of WSSV copies (Fig 2K). The protein levels of WSSV, as detected by the anti-VP26 antibody, were increased in hemocytes and intestine (Fig 2L). In general, these data indicated that *Mj*pIgR promoted the proliferation of WSSV in shrimp.

### *Mj*pIgR oligomerizes to a tetramer and is internalized into the cytoplasm of hemocytes after WSSV infection

**Fig 3:**
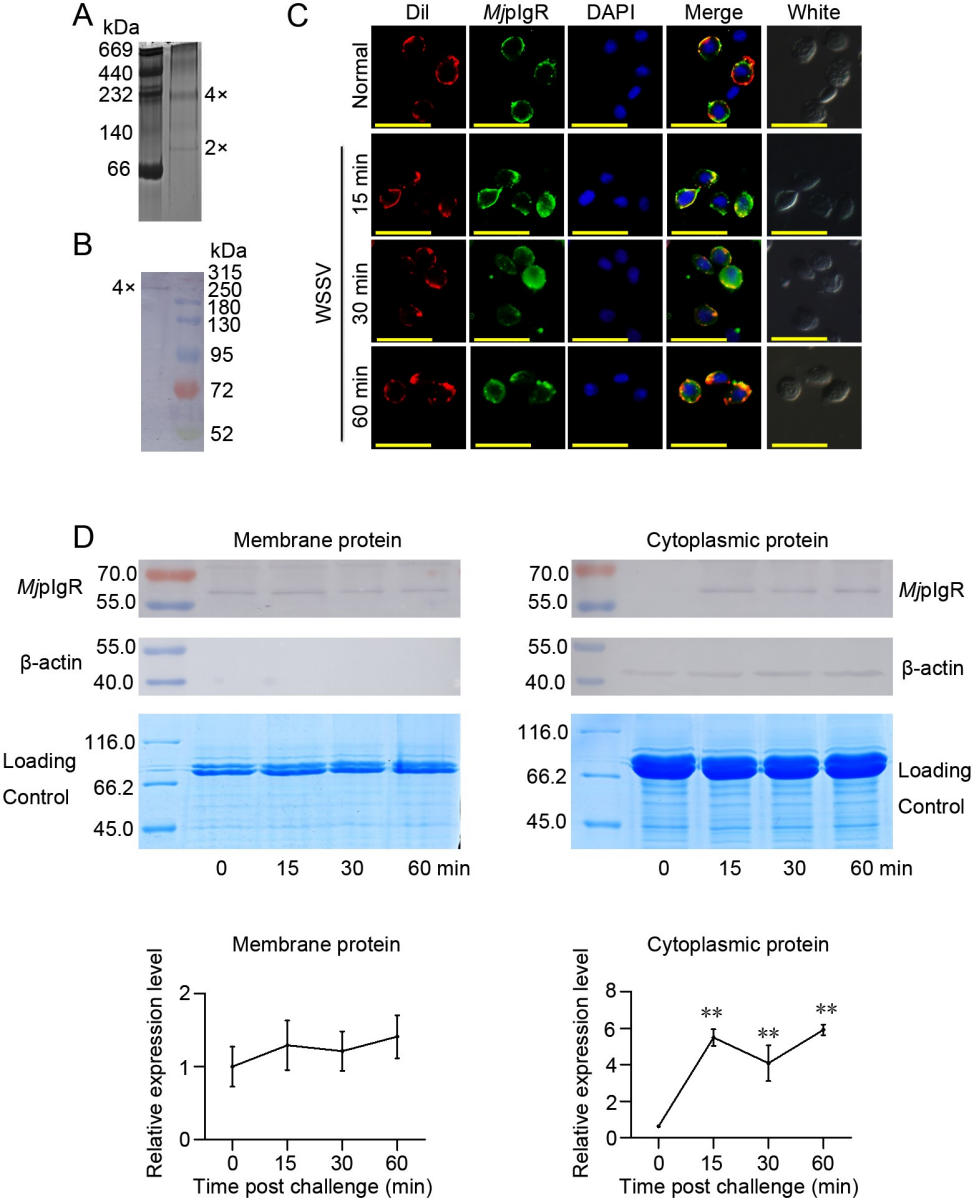
*Mj*pIgR oligomerized to tetramers and internalized into the cytoplasm of hemocytes in shrimp after WSSV infection. **A**, Native PAGE of r*Mj*pIgR. Purified r*Mj*pIgR was analyzed using native PAGE and stained with Coomassie blue. **B**, A tetramer of *Mj*pIgR was detected *in vivo* using western blotting after treatment of hemocytes with a crosslinker (BS3). Shrimp were injected with WSSV, after 30 min, the hemocytes were collected and treated with BS3. These hemocytes were homogenized and the extracted proteins were separated by SDS-PAGE. Western blotting was then performed using anti-*Mj*pIgR antibodies. **C**, *Mj*pIgR in hemocytes of shrimp was detected at 0 (untreated), 15, 30, and 60 min post WSSV injection. Scale bar = 20μm. **D**, Shrimp were challenged with WSSV and then membrane and cytoplasm proteins of the hemocytes were extracted. *Mj*pIgR in the membrane and cytoplasm of hemocytes was analyzed using western blotting at 0, 15, 30, and 60 min post-WSSV injection. The lower panels show the statistical analysis from three independent experiments. **, *p* < 0.01.

To analyze the possible mechanism of *Mj*pIgR in WSSV proliferation, *Mj*pIgR oligomerization and internalization were detected. The oligomerization of recombinant *Mj*pIgR (r*Mj*pIgR) was first analyzed using native PAGE, and the result showed that r*Mj*pIgR formed different oligomers *in vitro* (Fig 3A). Further studies showed that the native *Mj*pIgR formed a tetramer, determined by the molecular mass after WSSV challenge *in vivo* (Fig 3B). We performed immunocytochemistry to detect the subcellular localization of *Mj*pIgR using anti-*Mj*pIgR antibodies. Under normal conditions, *Mj*pIgR was mainly located on the cell membrane (Fig 3C top panels). After WSSV challenge, *Mj*pIgR gradually moved from surface to the cytoplasm as challenge time increased from 15 to 60 min (Fig 3C). These results suggested that the internalization of *Mj*pIgR might be associated with WSSV endocytosis. We further analyzed *Mj*pIgR in the membrane and cytoplasm of hemocytes using western blot. The results showed that the *Mj*pIgR level in membrane of hemocytes decreased slightly with increasing WSSV challenge time; however, its levels showed no significant differences between time points (Fig 3D left panels). The *Mj*pIgR level increased significantly in the cytoplasm of hemocytes after WSSV infection (Fig 3D right panels). These results suggested that the *Mj*pIgR was internalized from the membrane into the cytoplasm and this internalization might be related to WSSV endocytosis.

### *Mj*pIgR co-localized with WSSV in hemocytes

**Fig 4:**
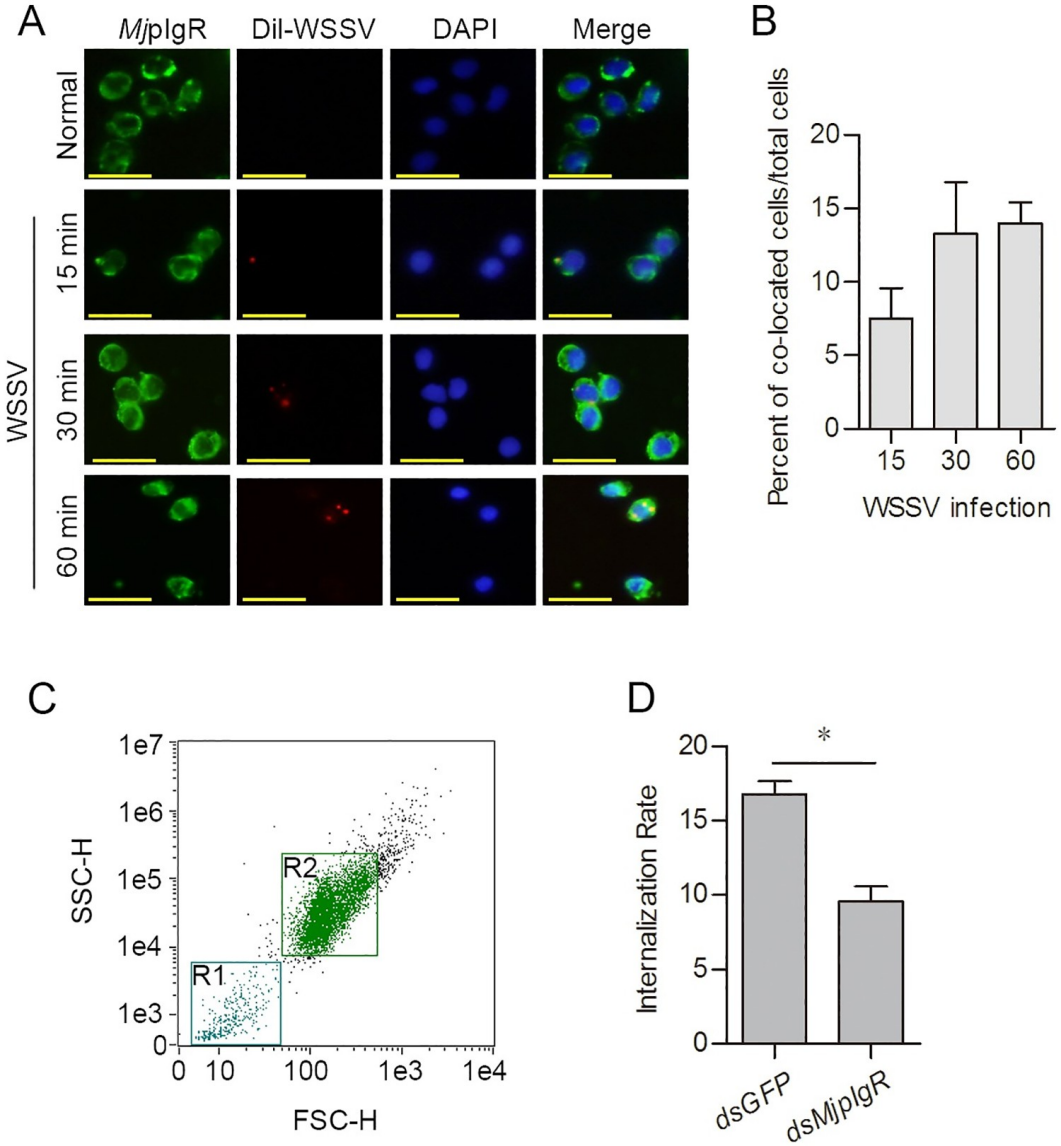
*Mj*pIgR co-localized with labeled WSSV. **A**, Immunocytochemistry was used to detect the co-localization of *Mj*pIgR and Dil-labeled WSSV virions in hemocytes. The hemocytes were collected at different time points (15, 30, and 60 min) post-WSSV injection. Scale bar = 20 μm. **B**, Statistical analysis of *Mj*pIgR-WSSV co-localized cells compared with total cells. **C**, Intact hemocytes (R2) were separated from incomplete fragments (R1) using flow cytometry. **D**, Flow cytometry was performed after RNAi of *Mj*pIgR to test the internalization rate of hemocytes. *, *p* < 0.05.

To determine whether the internalization of *Mj*pIgR is required for endocytosis of WSSV, an immunocytochemical assay was performed to detect the co-localization of *Mj*pIgR with WSSV. The co-localization of *Mj*pIgR with Dil-labeled WSSV was observed at 15 min post WSSV injection (Fig 4A) and the co-localization rate increased from 15 to 60 min, and the WSSV moved to perinuclear location at 60 min (Fig 4B). The results suggested that *Mj*pIgR might be a receptor for WSSV that is involved in endocytosis of WSSV in shrimp. To further confirm the results, flow cytometry was performed after RNA interference of *Mj*pIgR. The results showed that the internalization rate of WSSV particles decreased in hemocytes after RNAi of *Mj*pIgR (Fig 4C and 4D). These results suggested that endocytosis of WSSV required the internalization of *Mj*pIgR in shrimp hemocytes and that *Mj*pIgR might be a receptor for WSSV.

### *Mj*pIgR can independently render non-permissive cells (HEK 293T) susceptible to WSSV infection

**Fig 5:**
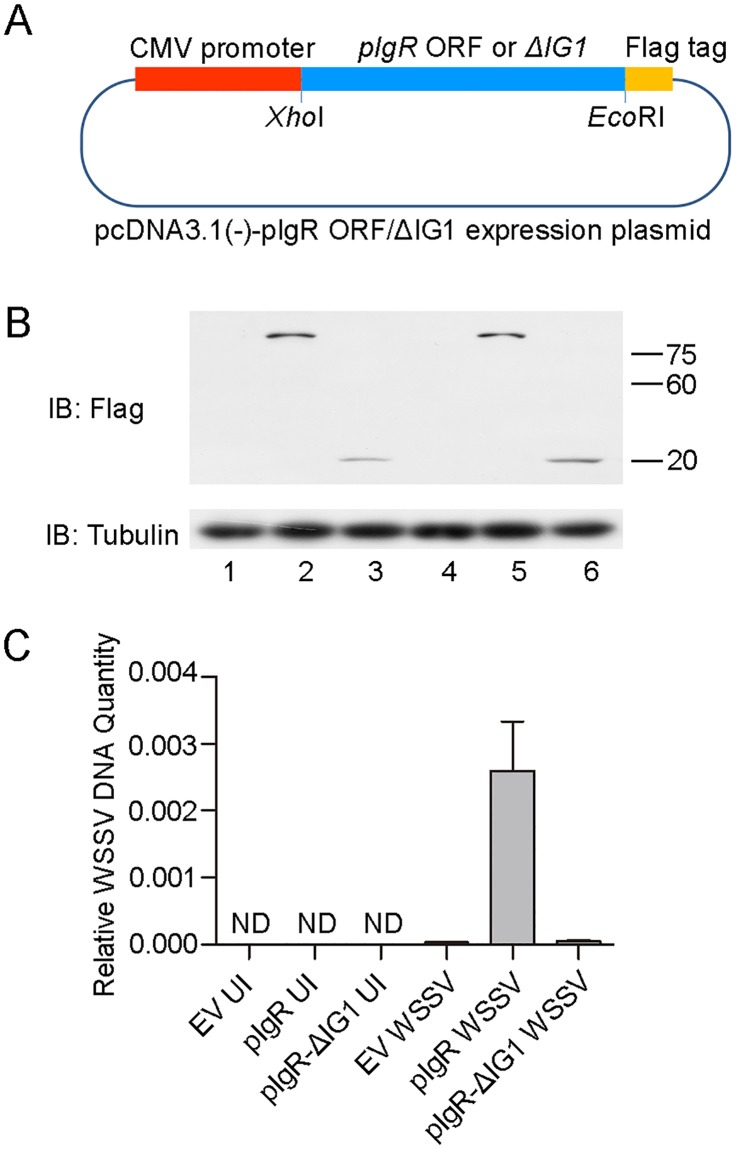
*Mj*pIgR is sufficient for gaining entry into non-permissive cells (HEK 293T) transfected with *Mj*pIgR expression plasmid. (A) pcDNA3.1(-)-pIgR map. (B) Western blot to analyze *Mj*pIgR expression in HEK 293T cells. Lane 1, Uninfected HEK 293T cells; lane 2, HEK 293T cells transfected with pcDNA3.1(-)-pIgR plasmids uninfected with WSSV; lane 3, HEK 293T cells transfected by pcDNA3.1(-)-pIgR-ΔIG1 plasmids uninfected with WSSV; line 4, empty vector infected with WSSV, lane 5. HEK 293T cells transfected by pcDNA3.1(-)-pIgR plasmids with WSSV infection; lane 6, HEK 293T cells transfected by pcDNA3.1(-)-pIgR-ΔIG1 plasmids with WSSV infection. (C) qPCR analysis of WSSV DNA in WSSV-infected HEK 293T Cells transfected with Empty Vector or pcDNA3.1(-)-pIgR/ΔIG1 expression plasmid. The qPCR results presented relative to Genomic DNA. ND: Not Detected; UI: Uninfected; EV: Empty Vector; pIgR: pIgR ORF fusion to human pcDNA3.1(-); pIgR-ΔIG1: the truncated IG1 domain of pIgR fusion to human pcDNA3.1(-).

To address whether *Mj*pIgR could independently facilitate WSSV entry, we assessed the level of WSSV entry in human HEK 293T cells upon *Mj*pIgR overexpression. The plasmids pcDNA3.1(-)-pIgR and pcDNA3.1(-)-pIgR-ΔIG1 were constructed to express *Mj*pIgR and pIgR-ΔIG1 (containing a truncating mutation of pIgR) in the non-permissive cells (human HEK 293T cells) (Fig 5A). Then HEK 293T cells were transfected with empty vector, pcDNA3.1(-)-pIgR or pcDNA3.1(-)-pIgR-ΔIG1, respectively. The cells were infected with WSSV for 1 hour or remained uninfected. After WSSV infection, the DNA of the cells was isolated and subjected to qPCR assay to detect the WSSV DNA. The expression of pIgR and pIgR-ΔIG1 were detected by western blot assays after transfection. The results showed that the pIgR and pIgR-ΔIG1 were successfully expressed on the cells (Fig 5B). The viral DNA could be detected in the pcDNA3.1(-)-pIgR transfected HEK 293T upon *Mj*pIgR overexpression, but not in pcDNA3.1(-)-pIgR-ΔIG1 transfected HEK 293T and empty vector transfected cells (Fig 5C). The results suggest that *Mj*pIgR serves as a receptor for WSSV entry and render the non-permissive cells (HEK 293T) susceptible to WSSV infection.

### *Mj*pIgR-SC binds to VP24 of WSSV

**Fig 6:**
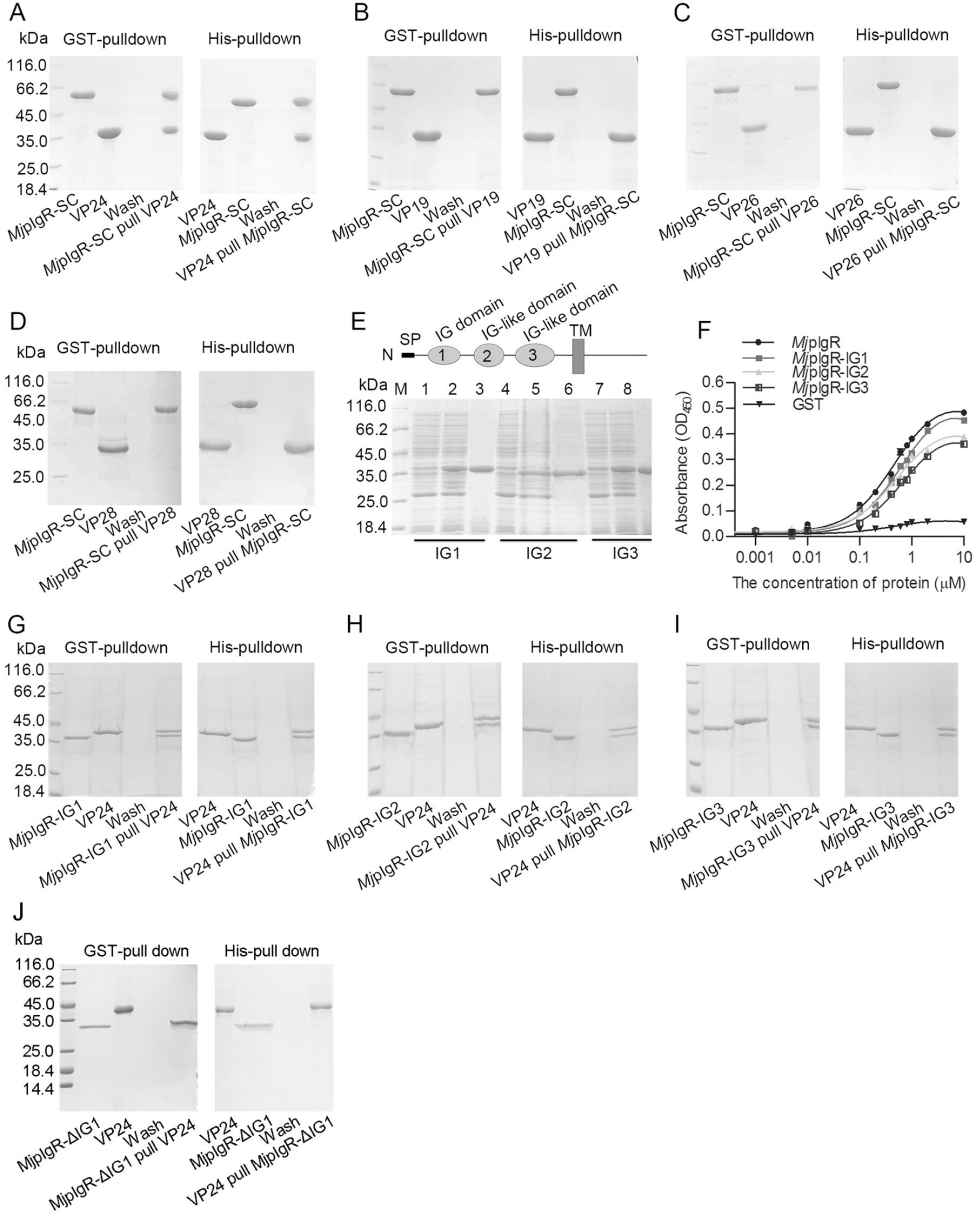
*Mj*pIgR binds to WSSV via its IG-like domains. **A**, The interaction of *Mj*pIgR-SC with VP24 was analyzed using GST- and His-Pulldown assays. **B**, The interaction of *Mj*pIgR-SC with VP19 was detected by GST- and His-Pulldown assays. **C**, GST- and His-Pulldown to analyze interactions of *Mj*pIgR-SC with His-VP26. **D**, GST- and His-Pulldown to detect interactions of *Mj*pIgR-SC with VP28. **E**, Recombinant expression and purification of IG1, IG2, and IG3 from *Mj*pIgR. Constructs pGEX4T-1-*Mj*pIgR-IG1, 2, or 3 were transformed into *E*. *coli*, and the expression of IG1-3 was induced by IPTG and analyzed by SDS-PAGE. Lanes 1, 4, and 7, total proteins from *E*. *coli* with pGEX4T-1-*Mj*pIgR-IG1, IG2, and IG3 without IPTG induction; lanes 2, 5, and 8, total proteins from the *E*. *coli* with IPTG induction; lanes 3, 6, and 9, purified recombinant *Mj*pIgR-IG1, -IG2, and IG3 proteins; lane M, protein molecular mass standard. **F**, ELISA assay to detect the binding of *Mj*pIgR to WSSV. (**G-I**) Each of the IG domains (IG1–3) was used in a pulldown assay, including GST pulldown (left panel) and His pulldown (right panel) to detect the interaction of the IG domains with VP24. **J**, Truncating mutation of IG1 domain of *Mj*pIgR and its interaction with VP24 of WSSV analyzed by GST and His pulldown assays.

To confirm whether *Mj*pIgR is the entry receptor for WSSV, a binding assay was performed. We first recombinantly expressed the extracellular domains of *Mj*pIgR (*Mj*pIgR-SC) and then detected the interaction of *Mj*pIgR-SC with the envelope proteins of WSSV using *in vitro* GST- and His-pulldown assays. VP19, VP24, VP26, and VP28 of WSSV were used for the analysis. The results showed that *Mj*pIgR-SC interacted with VP24 (Fig 6A), but had no interaction with VP19, VP26, or VP28 (Fig 6B, 6C and 6D). To identify which of the extracellular Ig domains of *Mj*pIgR plays a central role in the interaction, the three Ig domains were recombinantly expressed and purified from *E*. *coli*, separately (Fig 6E). The binding ability of *Mj*pIgR-IG1, *Mj*pIgR-IG2, and *Mj*pIgR-IG3 to WSSV particles was analyzed using an ELISA binding assay. The results showed that all the three Ig domains bound to WSSV (Fig 6F). We further analyzed the interaction of different Ig domains with VP24 using pulldown assays and results indicated that all three Ig domains could interact with VP24 (Fig 6G, 6H and 6I). However, the truncating mutation of Ig domain could not interact with VP24 (Fig 6J). Taken together, the above results suggested that *Mj*pIgR could interact with WSSV through VP24 as a cellular receptor of WSSV.

### Calmodulin interacts with *Mj*pIgR-In and is involved in endocytosis

**Fig 7:**
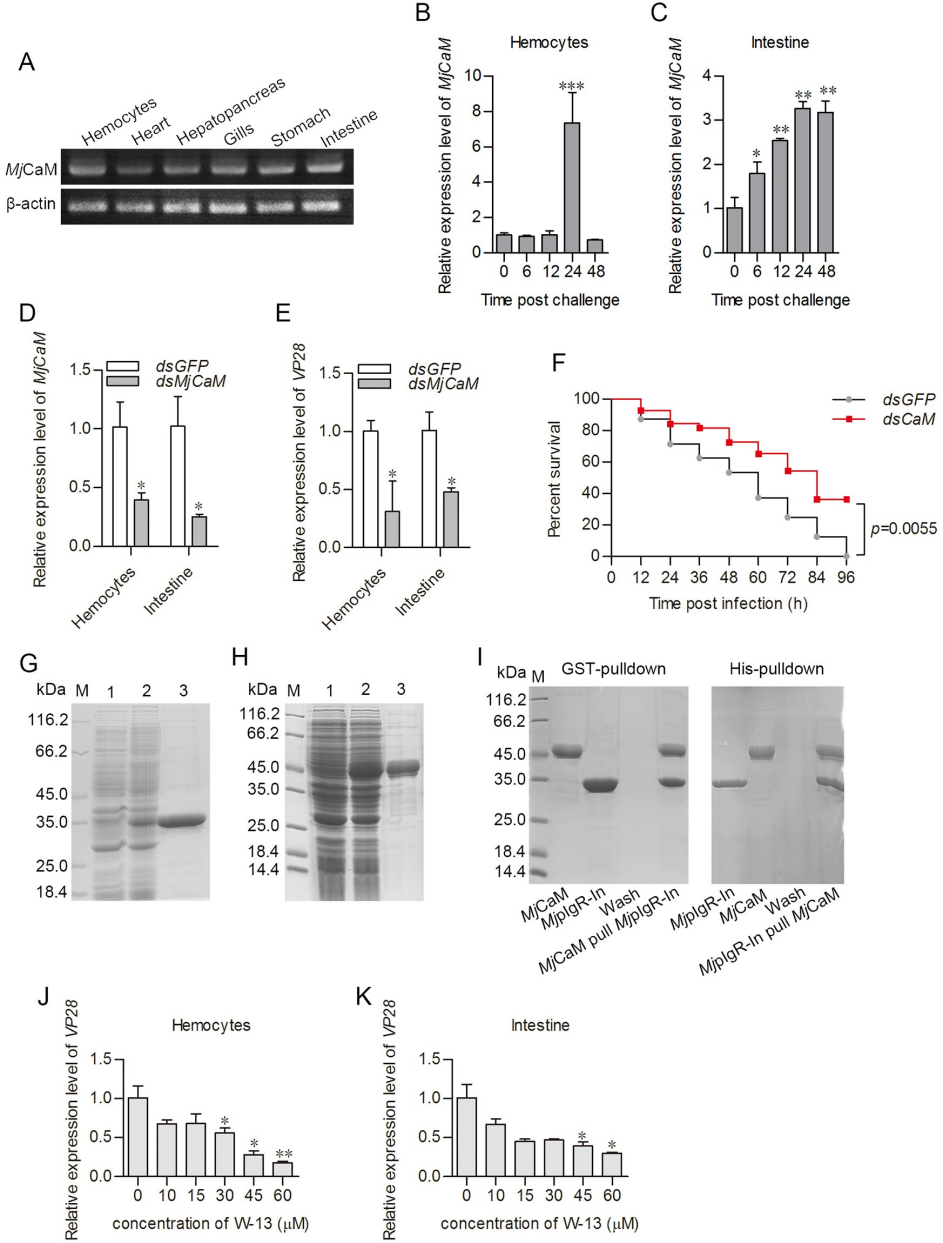
Calmodulin interacts with *Mj*pIgR-In and is involved in WSSV endocytosis. **A**, The tissue distribution of *MjCaM* mRNA. **B**, **C**, Expression levels of *MjCaM* in hemocytes and intestine, as detected by qPCR after WSSV infection. **D**, Efficiency of RNA interference of *MjCaM* in hemocytes and intestine. **E**, The *vp28* expression levels in *dsGFP* and *dsMjCaM* groups after WSSV challenge. **F**, Survival rates after WSSV infection in different interference groups. Significant differences were analyzed using the software GraphPad Prism 5.0. **G**, **H**, *Mj*pIgR-In and *Mj*CaM expression and purification from *E*. *coli*. Lane 1, total proteins from *E*. *coli* with pET32a-*MjpIgR-In* or pGEX4T-1-*MjCaM* without IPTG induction; lane 2, total proteins from the *E*. *coli* with IPTG induction; lane 3, purified recombinant *Mj*pIgR-In or *Mj*CaM protein. **I**, Interactions between His-tagged *Mj*pIgR-In and GST-tagged *Mj*CaM were detected using pull-down assays. **J**, **K**, Expression level of *vp28* in hemocytes and intestine treated with different concentrations of W-13. *, *p* < 0.05; **, *p* < 0.01; ***, *p* < 0.001.

The intracellular domain of human pIgR could interact with calmodulin, and calmodulin could interact with the clathrin heavy chain [17,42,43]. A calmodulin cDNA was cloned from *M*. *japonicus*, and named as *Mj*Calmodulin, (*Mj*CaM, GenBank Accession no. MH238441). *MjCaM* mRNA was distributed in all tissues tested (Fig 7A) and was upregulated by WSSV infection in hemocytes and intestine (Fig 7B and 7C). RNA interference was performed (Fig 7D) to explore the roles of *Mj*CaM in shrimp infected by WSSV. The *vp28* expression levels in hemocytes and intestine decreased in the *dsMjCaM*-injection group compared with that in the *dsGFP*-injection group (Fig 7E). The shrimp in the *dsMjCaM* group had a relatively higher survival rate compared with that of the controls (Fig 7F). The results indicated that *Mj*CaM promotes WSSV infection. To analyze the possible interaction of *Mj*pIgR with *Mj*CaM, the intracellular domain of *Mj*pIgR (*Mj*pIgR-In) and *Mj*CaM were expressed in *E*. *coli* (Fig 7G and 7H). The interaction between *Mj*pIgR-In and *Mj*CaM was analyzed using pull-down assays (Fig 7I). We found that *Mj*CaM could bind to *Mj*pIgR-In in *vitro*. A calmodulin antagonist, W-13, was also used for *Mj*CaM functional analysis in WSSV-infected shrimp. The results showed that in hemocytes and intestine, the *vp28* levels decreased in W-13 injected shrimp in concentration dependent manner (Fig 7J and 7K). The results suggested that *Mj*CaM facilitated WSSV proliferation and might be involved in the regulation of *Mj*pIgR internalization via their interaction.

### *Mj*pIgR-mediated WSSV endocytosis is clathrin-dependent

**Fig 8:**
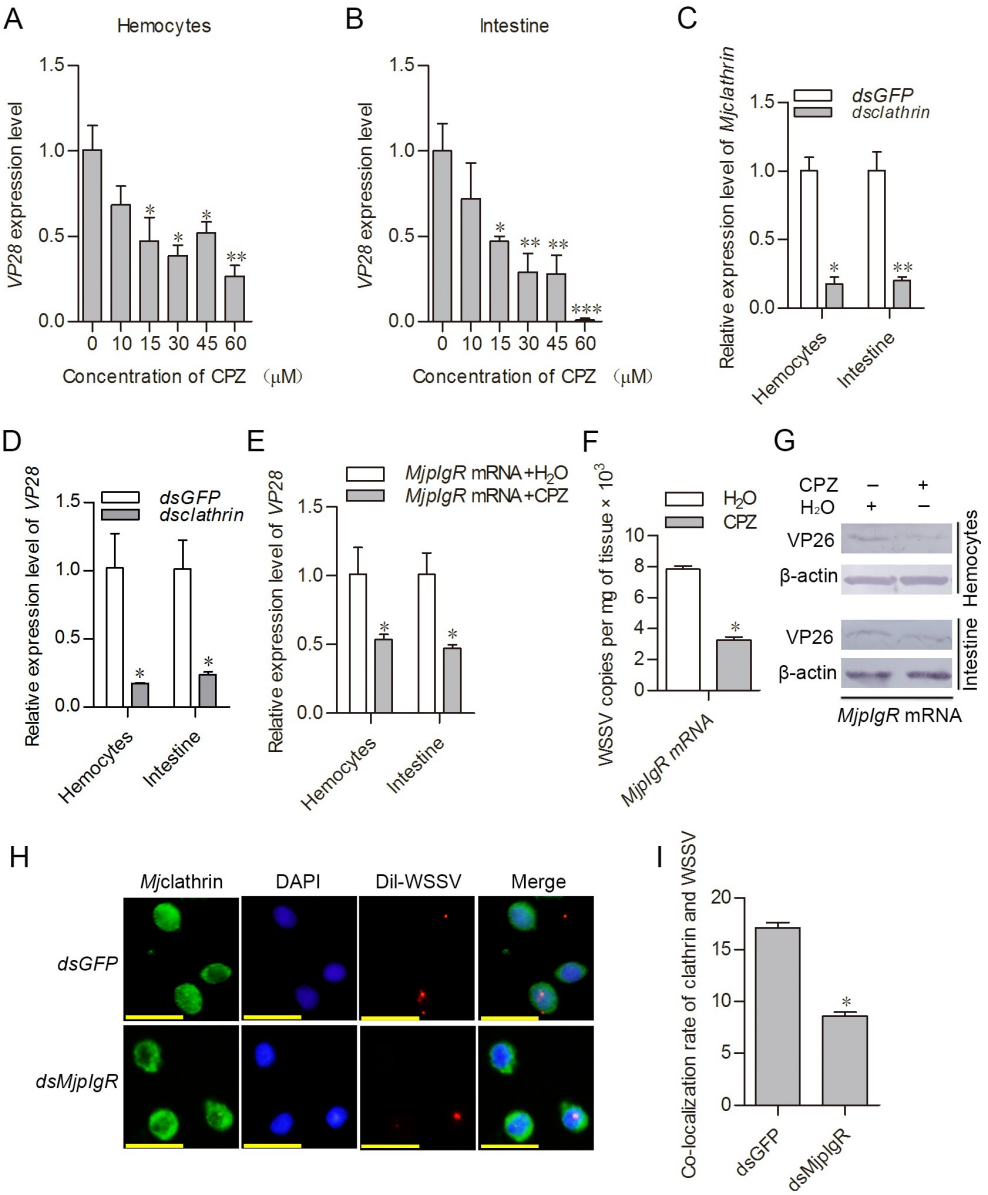
WSSV enters hemocytes via pIgR-CaM-clathrin-mediated endocytosis. **A**, **B**, CPZ, an inhibitor of clathrin-mediated endocytosis, was injected into shrimp, and the quantity of WSSV was detected using qPCR with *vp28* as an indicator. **C**, Efficiency of *Mjclathrin*-RNAi in hemocytes and intestine was determined using qPCR. **D**, The expression of WSSV *vp28* in *Mjclathrin*-knockdown shrimp infected with WSSV was detected via qPCR. **E**, The WSSV *vp28* expression level in *Mj*pIgR overexpression groups treated with H_2_O and CPZ. **F**, WSSV copies in the intestine of the H_2_O-injection group and CPZ-injection group. **G**, WSSV replication was detected by western blotting in CPZ-treated groups and controls. **H**, Immunocytochemistry was performed to detect the co-localization of WSSV and clathrin. Scale bar = 20μm. **I**, Statistical analysis of co-localization in the *dsGFP* and *dsMjpIgR* groups. For each group, three hundred hemocytes were counted under fluorescence microscopy and cells showing co-localization were recorded. *, *p* < 0.05; **, *p* < 0.01; ***, *p* < 0.001.

Viruses can hijack different cellular endocytic pathways for their internalization; among which, clathrin-mediated endocytosis is commonly used. Previously, we identified clathrin in the shrimp [40]. To determine whether the *Mj*pIgR-Calmodulin-mediated endocytosis of WSSV was clathrin-dependent, the dose-dependent blocking effect of virus infection by chlorpromazine (CPZ) was first determined. The results showed that CPZ caused a concentration-dependent decrease in the *vp28* expression level (Fig 8A and 8B). After RNAi of *Mj*clathrin (Fig 8C), the *vp28* expression level also declined significantly (Fig 8D). To confirm whether clathrin-mediated endocytosis was associated with *Mj*pIgR, the mRNA of *MjpIgR* was overexpressed, and then CPZ was injected into the overexpression group after WSSV infection, and *vp28* expression was detected. The results showed that the ability of *Mj*pIgR to promote *vp28* expression was blocked by CPZ injection (Fig 8E). The number of WSSV copies in the intestine also decreased compared with that in the control group (Fig 8F). The same results were obtained using western blotting analysis of WSSV VP26 levels (Fig 8G). To further confirm that the endocytosis of WSSV via *Mj*pIgR was clathrin-dependent, the co-localization between clathrin and WSSV particles was detected. In the *dsMjpIgR* group, co-localization of clathrin and WSSV was reduced (Fig 8H and 8I). Taken together, the results suggested that WSSV enters shrimp cells via pIgR-CaM-clathrin-mediated endocytosis.

### Adaptor protein complex AP-2 associated with the endocytosis

**Fig 9:**
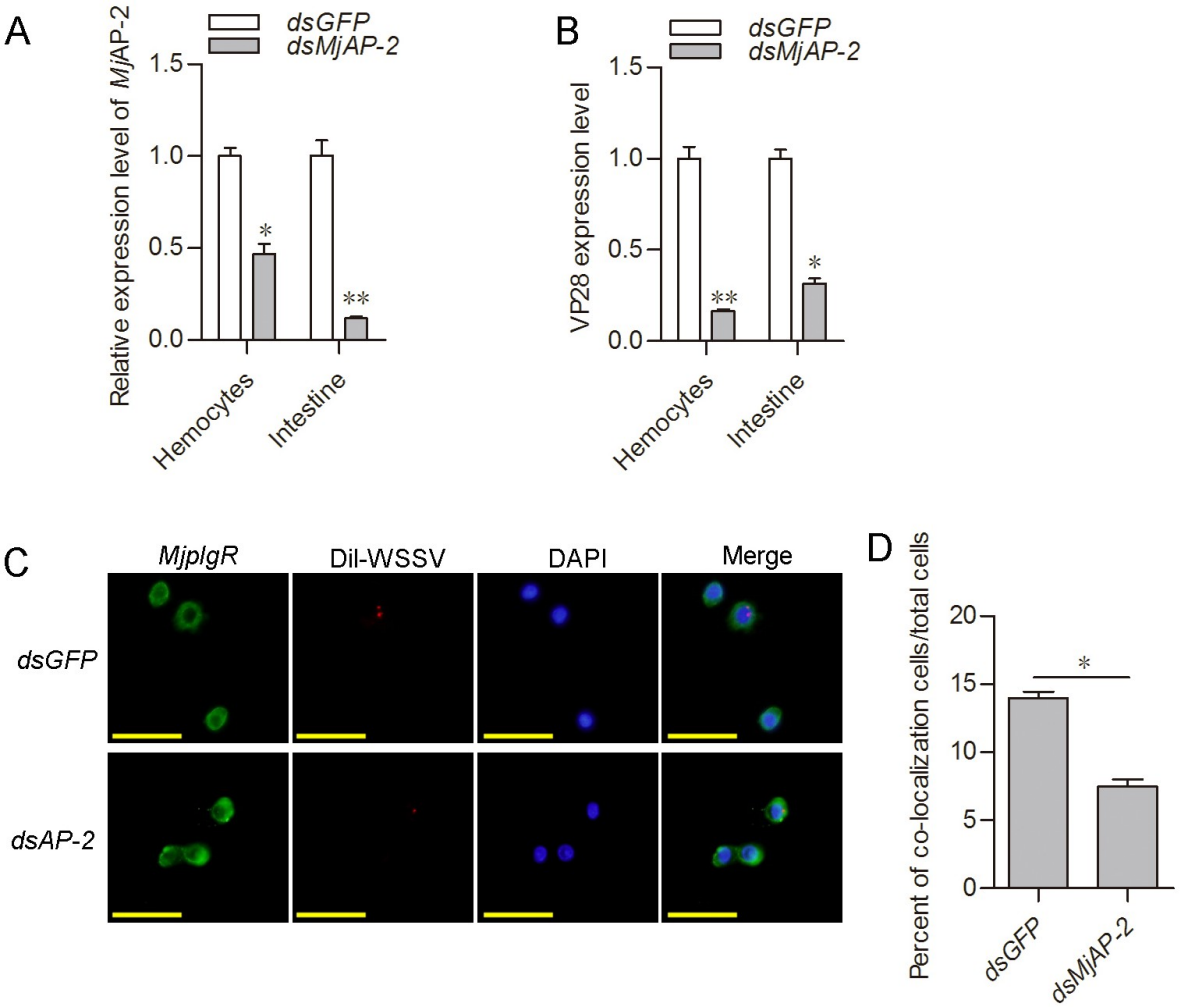
Adaptor protein complex AP-2 associated with the clathrin-mediated endocytosis. **A**, Efficiency of *AP-2* RNAi in hemocytes and intestine. **B**, *vp28* expression levels in hemocytes and intestine of *AP-2*-knockdown shrimp. **C**. Co-localization of *Mj*pIgR and Dil-labeled WSSV virions in hemocytes of *AP-2*-knockdown shrimp after WSSV infection for 1 h. Scale bar = 20 μm. **D**, Statistical analysis of *Mj*pIgR-WSSV co-localized cells compared to the control.

Clathrin-based endocytic pathways involve a variety of adaptor proteins. The adaptor protein complex AP-2 has been considered one of the core components of the clathrin-based endocytic machinery [44,45,46]. We also identified complex AP-2 in shrimp, including AP-2α, β, μ and σ in the shrimp. To confirm above result about WSSV entering shrimp cells via clathrin-mediated endocytosis, the AP-2α was knockdown by RNAi (Fig 9A), and WSSV replication and colocalization of *Mj*pIgR with WSSV were detected. The results showed that the WSSV replication declined (Fig 9B) and the colocalization decreased (Fig 9C and 9D) upon down regulation of endocytic pathway, suggesting that pIgR-CaM-clathrin-mediated endocytosis associated with the classical adaptor protein complex AP-2 in shrimp.

## Discussion

In the present study, we identified a key receptor, *Mj*pIgR, for WSSV entry and infection. The extracellular domain of *Mj*pIgR could interact with WSSV envelope protein VP24 and intercellular domain interacted with *Mj*CaM. *Mj*CaM recruited *Mj*clathrin, and AP-2 adaptor complex also associated with the viral entry. Therefore, WSSV entered host cells via the pIgR-CaM-clathrin endocytotic pathway.

The *Mj*pIgR sequence obtained from *M*. *japonicus* possesses three immunoglobulin domains and is a member of the IgSF. As cell adhesion molecules, IgSF is a large protein superfamily of cell surface or soluble proteins that are involved in the recognition, binding, or adhesion processes of cells [47]. By BLAST analysis, the IgSF member from *M*. *japonicus* was observed to be similar with fasciclin molecules, especially fasciclin III. However, by domain architecture comparison (S1 Fig), we found that Fasciclin I contains Fas 1 (Fasciclin-like) domains, Fasciclin II has Ig and FN3 (Fibronectin type 3) domains, and Fasciclin III possesses Ig or Ig-like domains, in addition to a transmembrane motif. Compared with polymeric immunoglobulin receptor (pIgR) (S2 Fig), the domain architecture of the IgSF member from *M*. *japonicus* is quite similar to vertebrate pIgRs. The phylogenetic analysis also showed the similarity of the IgSF member from *M*. *japonicus* with pIgR from vertebrates (S3 Fig). Therefore, we designated the IgSF from kuruma shrimp as a pIgR-like protein (*Mj*pIgR). In the present study, as a receptor for WSSV, *Mj*pIgR carrying WSSV particles enters hemocytes and induces a systemic infection in shrimp.

Most of the IgSF members, including pIgR, are type I transmembrane proteins, which comprise an extracellular domain (containing one or more Ig-like domains), a single transmembrane domain, and a cytoplasmic region [48]. These IgSF proteins can mediate adhesion through their N-terminal Ig-like domains, which usually bind other Ig-like domains of the same structure on the cell surface, or interact with other molecules, such as integrins and carbohydrates [49]. This suggested that the IgSF molecules could form homopolymers. In our study, we found that *Mj*pIgR formed tetramer *in vivo* and interacts with VP24 of WSSV.

Several classes of molecules are exploited as receptors by diverse groups of viruses, including sialic acid moieties [50] and integrins [51,52]. In particular, many IgSF proteins, such as pIgRs, have been identified as viral receptors [7,53], such as the HIV receptor (T-cell surface glycoprotein CD_4_) [25], main rhinovirus receptors (intracellular adhesion molecule-1), and poliovirus receptor and adeno-associated virus receptors [30]. In our study, we found that *Mj*pIgR interacted with VP24 of WSSV, and was used by WSSV as a receptor for its entry into cells.

As one of the key processes of infection, DNA virus entry into host cells requires distinct cellular processes, including attachment to receptors; signaling; movement of the virus on the cell surface; endocytic uptake and trafficking; and uncoating of the genome, followed by replication, and, finally, particle assembly and release [54]. Usually, virus entry starts with binding to attachment factors, followed by association with receptors. The attachment factors merely bind the viruses and thus help to concentrate the viruses on the cell surface. The virus receptors can trigger changes in the virus, induce cellular signaling, promote endocytosis, and accompany the virus into the cell. However, the differentiation of attachment factors and receptors is often difficult in practice because both of them contribute to effective infection. Many viruses have evolved multi-step attachment processes, and a requirement for more than one receptor molecule is not uncommon. An extreme example is hepatitis C virus (HCV), which requires more than ten molecules for cell entry [7]. For WSSV infection, Verbruggen et al. (2016) summarized the possible receptors or receptor complexes for WSSV, which include Chitin-binding protein, glucose transporter 1, integrin, calreticulin, and C-type lectins (such as *Mj*scCL, *Mj*LecA-C) [55]. However, among the reported WSSV receptors, few molecules are genuine transmembrane proteins. We inferred that certain soluble molecules of the “receptors”, such as C-type lectin (*Mj*svCL, *Mj*LecA-C), Rab proteins, and Chitin-binding proteins, might be the attachment factors for WSSV, and that membrane proteins such as integrins [35], and scavenger receptors [40] were the receptors for WSSV. In the present study, our results might hint that *Mj*pIgR is not the only receptor involved in viral entry. As shown in Fig 2, the difference of survival rate of *Mj*pIgR-silenced shrimp and the control group although shows significant, a moderate improvement in survival of the two groups is observed (Fig 2G). This might suggest that there are other receptors and pathways may function in WSSV adhesion/entry processes. To answer the questions, we knocked down the expression of *β-Integrin* (a previously reported WSSV receptor) in shrimp, WSSV replication and survival rate were analyzed (S5 Fig). The results showed that after knockdown of *β-Integrin* (S5A Fig), WSSV replication declined (S5B Fig) and survival rate of the shrimp was higher than that of control group (S5C Fig). Comparing the results of *MjpIgR* (Fig 2G) and *β-Integrin* knockdown (S5C Fig) experiments, a similar moderate survival improvement was observed. These results suggested that like other viruses, WSSV has evolved multi-step attachment processes, and a requirement for more than one receptor in its infection. To date, however, no IgSF member has been reported to be a WSSV receptor. Therefore, this is the first report of a WSSV IgSF receptor.

Receptors play a crucial role in determining the cell specificity and tissue tropism of viruses. WSSV exhibits a much broader cell tropism and can infect most cell types from organs of ectodermal and mesodermal origin, including those of the epidermis, gills, foregut, hindgut, lymphoid organ, muscle, heart, and gonads [56]. As a transmembrane receptor, *Mj*pIgR was ubiquitously distributed in shrimp. The wide distribution of *Mj*pIgR corresponded with WSSV’s broad cell tropism in shrimp. To ascertain whether *Mj*pIgR is the WSSV receptor, we detected the WSSV entry in non-permissive cells (HEK 293T) with *Mj*pIgR overexpression. The result showed that *Mj*pIgR can independently render non-permissive cells (HEK 293T) susceptible to WSSV infection, and suggesting that *Mj*pIgR is one of the receptors of WSSV infection (Fig 5B and 5C).

After binding to receptors on the cell surface, the enveloped virus can either penetrate the membrane directly by lipid fusion and membrane perforation, or enter the host cell by endocytosis [41]. How does the endocytosis take place? The extracellular signal should be transferred to the cell cytoplasm by the receptor. The C-terminal intracellular domains of IgSF members often interact with cytoskeletal or adaptor proteins. This interaction can lead to the extracellular interaction signal being transmitted to the inside of the cells, which enables IgSF proteins to function in a wide range of biological processes [47]. In the present study, we identified that the intracellular domain of *Mj*pIgR interacts with calmodulin (*Mj*CaM). Several studies have reported that calmodulin could interact with clathrin [42,57,58]. In the immunocytochemical analysis, the membrane *Mj*pIgR moved to the cytoplasm. In addition, *Mj*pIgR and clathrin colocalized with WSSV in the cells. This suggested that the endocytosis of WSSV was pIgR-calmodulin-clathrin dependent. The AP-2 adaptor complex is a multimeric protein that has been considered one of the core components of clathrin-mediated endocytosis. We also knocked down the AP-2α, a large subunit of the complex, and found that WSSV replication and co-localization of *Mj*pIgR with VP28 were decreased (Fig 9). The results suggested that AP-2 also associated with clathrin-mediated endocytosis in shrimp.

**Fig 10:**
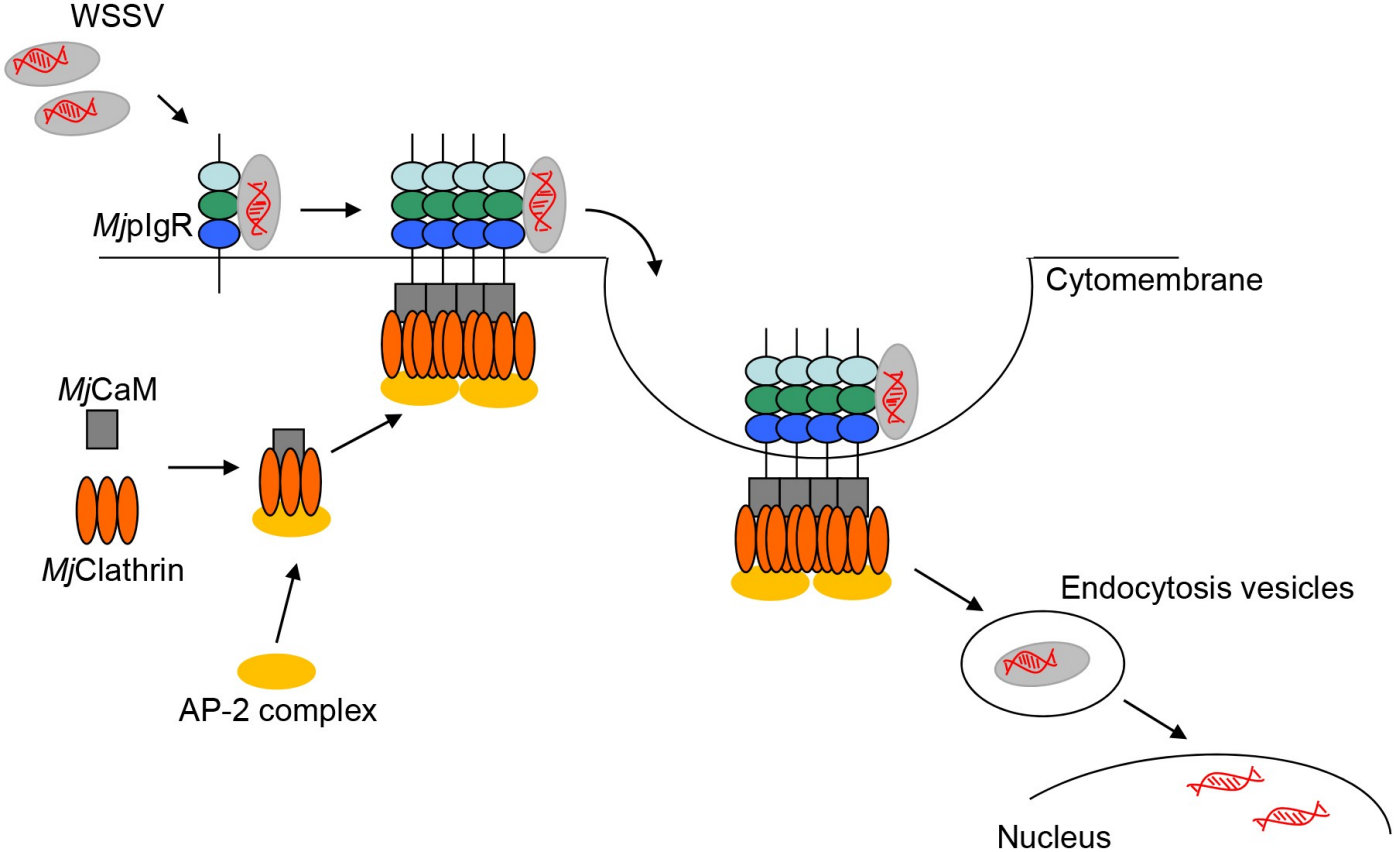
Schematic of *Mj*pIgR in promoting WSSV endocytosis as a transmembrane receptor. WSSV bind to the extracellular domains of *Mj*pIgR and activated receptor-mediated endocytosis. *Mj*pIgR was oligomerized and intracellular domain of *Mj*pIgR interacts with *Mj*CaM, and *Mj*CaM interacts with *Mj*clathrin. AP-2 adaptor complex also involves in the clathrin mediated endocytosis. *Mj*pIgR with bound WSSV internalized in the pIgR-CaM-Clathrin endocytosis pathway.

In conclusion, WSSV enters host cells by attachment to the primary receptor, *Mj*pIgR, on the cell membrane, a process that might require other attachment factors or coreceptors. The binding WSSV with the receptor induces oligomerization of the receptor to tetramers and the signal is transferred to the cell cytoplasm, resulting in the intracellular domain of *Mj*pIgR interacting with calmodulin. This further induces the interaction of calmodulin with clathrin, finally resulting in endocytosis of WSSV into the host cells (Fig 10). The trafficking, penetration, and genome uncoating of the incoming WSSV in the host cell require further study.

## Materials and methods

### Animals

Healthy *M*. *japonicus* (9 g to 12 g each) were purchased from a seafood market in Jinan City, Shandong province, China. The shrimp were acclimated for 48 h in an aerated aquarium with artificial seawater at about 24 °C. The salinity of the seawater was maintained between 24‰ (w/v) to 26‰. Animals were randomly selected for the following experiments.

### cDNA cloning and sequence analysis

The full-length sequence of *Mj*pIgR was obtained through transcriptome sequencing using different tissues from infected shrimps. We used the NCBI database to determine the sequence homology (http://blast.ncbi.nlm.nih.gov/Blast.cgi). The amino acid sequence, theoretical molecular weight, and isoelectric point of *Mj*pIgR were analyzed using the online server (http://web.expasy.org/translate/). A domain prediction tool (SMART: http://smart.embl-heidelberg.de/) was used to analysis the protein domain architecture.

### WSSV challenge and tissue collection

The WSSV inoculum was extracted based on the previously described method and the quantitative real-time PCR (qPCR) was used for viral quantification [59]. Each shrimp was injected with 50 μl of WSSV virions (1 × 10^5^) from the viral infection. The same volume of sterile phosphate-buffered saline (PBS) (140 mM NaCl, 2.7 mM KCl, 10 mM Na_2_HPO_4_, 1.8 mM KH_2_PO_4_, pH 7.4) was injected into the control groups. Hemocytes were extracted from shrimp using a sterile syringe with anticoagulant buffer (450 mM NaCl, 10 mM KCl, 10 mM EDTA, 100 mM HEPES, pH 7.45) and then the hemolymph was discarded after centrifugation at 800 × *g* for 6 min at 4 °C, while the other tissues were dissected with scissors and forceps on ice for RNA or protein extraction.

### RNA extraction, cDNA synthesis, and DNA and protein extraction

Total RNA was isolated from hemocytes and different organs (heart, hepatopancreas, gills, stomach, and intestine) of shrimp using the TRIpure Reagent (Bioteke, Beijing, China). First-strand cDNAs were synthetized using a cDNA Synthesis Kit (M-MLV version; Takara, Dalian, China). Genomic DNA was extracted using a genomic DNA Extraction Kit (Toyobo, Osaka, Japan). Protein samples from different organs and hemocytes were homogenized separately in radio-immunoprecipitation assay (RIPA) buffer (50 mM Tris-HCl, 150 mM NaCl, 0.1% SDS, 0.5% Nonidet P-40, 1 mM EDTA, 0.5 mM PMSF, pH 7.5). The tissue homogenate was centrifuged at 12000 × *g* for 10 min at 4 °C to collect the supernatant for further analysis.

### Recombinant expression, purification, and antiserum production of *Mj*pIgR

The specific primers *Mj*pIgR-EX-F and *Mj*pIgR-EX-R (Table 1) were used to amplify the extracellular fragment of *Mj*pIgR. The PCR procedure was as follows: One cycle at 94 °C for 3 min; 35 cycles at 94 °C for 30 s, 54 °C for 30 s, and 72 °C for 90 s; and one cycle at 72 °C for 10 min. The PCR fragments were digested with restriction enzymes *Xho*I and *Eco*RI, and then ligated into the pGEX-4T-1 vector (GE Healthcare, Piscataway, NJ, USA). The recombinant plasmid was transformed into *Escherichia coli* Rosseta (DE3) cells. GST-tagged *Mj*pIgR recombinant expression was induced with 0.5 mM isopropyl-β-D-thiogalactopyranoside (IPTG) at 37 °C for 4 h. The *Mj*pIgR inclusion bodies were washed two times with Buffer A (50 mM Tris-HCl, 5 mM EDTA, pH 8.0) and then three times with Buffer B (50 mM Tris-HCl, 5 mM EDTA, 2 M urea, pH 8.0), and the precipitate was collected by centrifugation at 12000 × *g* for 10 min at 4 °C. Denaturing solution (0.1 M Tris-HCl, 10 mM DL-Dithiothreitol, 8 M urea) was added to dissolve the precipitate. The solution was shaken at 37 °C for 30 min and then the supernatant was collected after centrifugation at 12000 × *g* for 10 min at 4 °C. The *Mj*pIgR was refolded in TBS buffer (150 mM NaCl, 3 mM EDTA, 50 mM Tris-HCl, pH 8.0) for 48 h at 4 °C. The protein was purified using an affinity chromatography with GST-resin (GenScript, Nanjing, China) according to the manufacturer’s instructions. Rabbit antiserum against *Mj*pIgR was prepared following a previously reported method [60]. The three IG domains of *Mj*pIgR and *Mj*pIgR-ΔIG1 were also expressed in *E*. *coli* and purified for pulldown analysis.

**Table 1:** Sequences of the primers used in this research.

Primer	Sequence (5’-3’)
**Recombinant expression**	
*Mj*pIgR-SC-EX-F	TACTCAGAATTCGACGCAGACATTATCGTG
*Mj*pIgR-SC-EX-R	TACTCACTCGAGTGAAAGCCGCACTTTGTA
*Mj*pIgR-IG1-EX-F	TACTCAGAATTCGACGCAGACATTATCGTG
*Mj*pIgR-IG1-EX-R	TACTCACTCGAGCTTCACAGTTACGATCAC
*Mj*pIgR-IG2-EX-F	TACTCAGAATTCGAGACACCAGTTAAGTGT
*Mj*pIgR-IG2-EX-R	TACTCACTCGAGAGGCACAGTGAGGGTCTT
*Mj*pIgR-IG3-EX-F	TACTCAGAATTCAGCACTTACTACCAGATT
*Mj*pIgR-IG3-EX-R	TACTCACTCGAGTGAAAGCCGCACTTTGTA
*Mj*pIgR-ORF-F	TACTCAGAATTCATGTGCGAGTGTTCCTTTGTG
*Mj*pIgR-ORF-R	TACTCACTCGAGCTACTCCTTTTTCTCGTCCTCCCG
*Mj*pIgR-ΔIG1-EX-F	TACTCAGAATTCGACGCAGACATTATCGTG
*Mj*pIgR-ΔIG1-EX-R	TACTCACTCGAGTCCAGGATGGACATCATC
*Mj*pIgR-In-EX-F	TACTCAGAATTCCGGTATCGCCAGATGTTC
*Mj*pIgR-In-EX-R	TACTCACTCGAGTGTCACGGTTCCAACAAT
*Mj*calmodulin-EX-F	TACTCAGGATCCATGGCGGATCAGCTGACCGAA
*Mj*calmodulin-EX-R	TACTCAGTCGACCTTCGAGGTCATCATCGTGAC
**(q)RT-PCR**	
*Mj*pIgR-RT-F	AAGTGTTAGCCGACAGGTTG
*Mj*pIgR-RT-R	CTAAGATTTGTCAGACGCACC
*Mj*clathrin-RT-F	ATTTGATAGAGTTTCGTCG
*Mj*clathrin-RT-R	GCAGGTCGTAGCAGTGGA
*Mj*calmodulin-RT-F	GGACGCTGACGGTAATG
*Mj*calmodulin-RT-R	GTGGTGGCTGCTTGATA
Actin-RT-F	AGTAGCCGCCCTGGTTGTAGAC
Actin-RT-R	TTCTCCATGTCGTCCCAGT
β-integrin-RT-F	CACATCAGCGTGACCTACA
β-integrin-RT-R	GACGAAAGAACCGAAACC
AP2-RT-F	GCTAAAGAAGAAGAGTGGGAGA
AP2-RT-R	GCAGGTGGAGTTGACAGG
VP28-RT-F	AGCTCCAACACCTCCTCCTTCA
VP28-RT-R	TTACTCGGTCTCAGTGCCAGA
**RNAi**	
*dsMj*pIgR-F	GCGTAATACGACTCACTATAGGCACAAGGAAGGAGTGGAGGTA
*dsMj*pIgR-R	GCGTAATACGACTCACTATAGGGGCGGTAACAATAATCAGCA
*ds*GFP-F	TAATACGACTCACTATAGGGGGGTGGTCCCAATTCTCGTGGAAC
*ds*GFP-R	TAATACGACTCACTATAGGGCTTGTACAGCTCGTCCATGC
*dsMj*clathrin-F	GCGTAATACGACTCACTATAGGCCCAACGCTGGTTATGCT
*dsMj*clathrin-R	GCGTAATACGACTCACTATAGGTGACCTGACCGCCTCTAC
*dsMj*calmodulin-F	GCGTAATACGACTCACTATAGGTTGTGATACATACACACA
*dsMj*calmodulin-R	GCGTAATACGACTCACTATAGGCGCGGTCACTTCACTTCG
*ds*β-integrin-F	GCGTAATACGACTCACTATAGGCCTGGACAAGAACTCGG
*ds*β-integrin-R	GCGTAATACGACTCACTATAGGCCTGGATAGCACGCACA
*ds*AP2-F	GCGTAATACGACTCACTATAGGGAACCTCCTTTCTTCCAACA
*ds*AP2-R	GCGTAATACGACTCACTATAGGACACGCAACCCTTATACTCA
**Overexpression of *Mj*pIgR in nonpermissive cells**	
pIgR-pcDNA3.1(-)-F	CTACCTCGAGATGTGCGAGTGTTCCTTTGTGC
pIgR-pcDNA3.1(-)-R	GACTGAATTCCTACTCCTTTTTCTCGTCCTCCCG
pIgR-ΔIG1-pcDNA3.1(-)-R	GACTGAATTCTCCAGGATGGACATCATCGTAAAC
WSSV VP28-RT-F	CTCCGCAATGGAAAGTCTGA
WSSV VP28-RT-R	GGGTGAAGGAGGAGGTGTT
Genomic DNA F	CCAACAAGTGTCTCCTCCAAAT
Genomic DNA R	AATCTCCTCAGGGATGTCAAAGT

### Western blotting

The tissue supernatants extracted from hemocytes and other organs (heart, hepatopancreas, gills, stomach, intestine) were resuspended in 200 μl of PBS, then 100 μl of SDS-PAGE Sample Loading Buffer (2% SDS, 0.1% bromophenol blue, 10% glycerin, 14.4 mM 2-Mercaptoethanol MCH, 50 mM Tris-HCl, pH 6.8) was added. The mixtures were centrifuged at 12000 × *g* for 1 min to collect the supernatants after treatment in a boiling water bath for 5 min. The proteins were separated by 12.5% SDS-PAGE and transferred onto a nitrocellulose membrane using Transfer Buffer (25 mM Tris, 20 mM Glycine, 0.037% SDS, 20% ethyl alcohol). After blocking with 3% nonfat milk diluted in TBST buffer (150 mM NaCl, 3 mM EDTA, 0.1% Tween-20, 50 mM Tris-HCl, pH 8.0) for 1 h, the membrane was incubated with antiserum against *Mj*pIgR (1:200 dilution in blocking milk solution) for 4 h at room temperature. The membrane was washed three times with TBST and then incubated with horseradish peroxidase (HRP)-conjugated goat anti-rabbit antibodies at 1:10,000 dilution in blocking reagent (ZSGB Bio, Beijing, China). The membrane was finally washed by TBST and TBS three times, respectively. Target bands were visualized via the colorimetric reaction by adding 10 ml reaction media (1 ml 4-chloro-1-naphthol and 6 μl H_2_O_2_, diluted in TBS). Western blotting bands were digitalized and statistic analyzed by Image J.

### Tissue distribution and expression profiles of *Mj*pIgR

The tissue distribution of *MjpIgR* mRNA was determined by semi-quantitative reverse transcription-PCR (RT-PCR) using primers *Mj*pIgR-RT-F and *Mj*pIgR-RT-R (Table 1). The β-actin gene was used as the internal control with primers β-actin-RT-F and β-actin-RT-R. The PCR procedure consisted of an initial incubation at 94 °C for 3 min; followed by 26 or 30 cycles of 94 °C for 30 s, 54 °C for 30 s, and 72 °C for 30 s; followed by 72 °C for 10 min. The PCR products were analyzed using agarose gel electrophoresis (1.2% agarose). Correspondingly, the tissue distribution at protein level was analyzed using western blotting. Anti-β-actin antibodies prepared in our laboratory were used for internal protein normalization.

Quantitative real-time PCR (qPCR) was performed to determine the expression profiles of *MjpIgR* mRNA after WSSV challenge using the above primers. The CFX96 Real-Time System (Bio-Rad, USA) was used to carry out the following PCR procedures: 95 °C for 10 min; 40 cycles at 95 °C for 15 s, 60 °C for 50 s, and reading at 72 °C for 2 s; and then a melting period from 65 °C to 95 °C. The data obtained were analyzed using the cycle threshold (2^−ΔΔCT^) method, as previously described [61]. The results were expressed as the mean ± SD from three independent repeats and significant differences in Student’s *t*-test were accepted at *p* < 0.05. Expression profiles of *Mj*pIgR were analyzed by western blotting at different infection times (0, 12, 24, 36, and 48 h) corresponding to the mRNA level.

### RNA interference and antibody blocking assays

Gene-specific primers *dsMj*pIgR-F and *dsMj*pIgR-R, linked to the T7 promoter (Table 1), were used to amplify a partial *Mj*pIgR cDNA fragment. The PCR products acted as the templates for double-stranded RNA (dsRNA) synthesis using T7 RNA polymerase (Fermentas, Burlington, Canada), following the manufacturer’s instructions. The *dsGFP* (Green fluorescent protein) coding region, serving as a control, was synthesized using primers *ds*GFP-F and *ds*GFP-R (Table 1). For the RNA interference assay, 50 μg of dsRNA was injected into shrimp and then another 50 μg of dsRNA was injected 12 h after the first injection. The efficiency of RNA interference was assessed at 24 h using qPCR. Similar method was used for knockdown of *Mj*clathrin, *Mj*calmodulin, *Mj*β-integrin and *Mj*AP-2α.

The pre-serum of rabbit and anti-*Mj*pIgR serum were purified as described previously [62]. Each shrimp was injected with 30 μg of purified antibodies for 2 h and then WSSV was injected into shrimp for 24 h. The *vp28* expression level was detected in hemocytes and intestine using qPCR.

### Survival assay

The survival rate was analyzed after RNAi of *MjpIgR* in shrimp challenged by WSSV. Shrimp were divided into two groups: The *dsGFP* group and the *dsMjpIgR* group. After RNAi for 24 h, WSSV particles (1 × 10^5^) were injected into two groups of shrimp separately. The two groups were monitored every half-day by counting the numbers of dead shrimp.

### *Mj*pIgR overexpression

To detect the function of *Mj*pIgR, an overexpression assay was performed. The *MjpIgR* open reading frame (ORF) was amplified using primers *Mj*pIgR-ORF-F and *Mj*pIgR-ORF-R (Table 1). The PCR fragments were then ligated into vector pET-32a(+), which contains a T7 promoter. Thereafter, the recombinant plasmid was used for mRNA synthesis and capping, as previous described [40]. The mRNA from empty pET-32a(+) vector was used as a control. Each group was injected with 100 μg mRNA for 24 h and the overexpression efficiency was detected using *Mj*pIgR antibodies. Later, WSSV particles were injected into the shrimp for additional 24 h. The RNA, DNA, and protein were extracted from different tissues to evaluate the quantity and copies of WSSV.

### pcDNA3.1(-)-pIgR construction and overexpression of WSSV in non-permissive cells

To qualify as a bona fide receptor of *Mj*pIgR for WSSV entry, the WSSV DNA was detected in *Mj*pIgR overexpressed non-permissive cell type (human HEK 293T cells). To construct the plasmid pcDNA3.1(-)-pIgR for expression of pIgR, the ORF of pIgR was amplified using the primers pIgR-pcDNA3.1(-)-F and pIgR-pcDNA3.1(-)-R (Table 1) and cloned into the *Xho*I and *Eco*RI restriction sites of the plasmid pcDNA3.1(-) vector. The truncated mutation of IG1 domain (named pIgR-ΔIG1) was also amplified with pIgR-pcDNA3.1(-)-F and pIgR-ΔIG1-pcDNA3.1(-)-R (Table 1) and cloned into pcDNA3.1(-)-pIgR-ΔIG1 plasmid.

HEK 293T cells were seeded in 24-well-plate one day before transfection. The pcDNA3.1(-)-pIgR expression plasmid, pcDNA3.1(-)-pIgR-ΔIG1 expression plasmid or empty vector was transfected into the HEK 293T cells using Lipofectamine 2000 (invitrogen) transfection reagent. Twenty-four hours later, WSSV was added into the cells and incubated at 37°C for 1 h. Then the cells were extensively washed with PBS twice to remove uninfected virus particles. Subsequently, the DNA of the cells was isolated using Dneasy Blood & Tissue Kit (QIAGEN), and subject to qPCR assay to detect the WSSV DNA. The primers used in qPCR assay is WSSV VP28-RT-F and WSSV VP28-RT-R; genomic DNA F and genomic DNA R.

### Immunocytochemical analysis

To detect the distribution and translocation of *Mj*pIgR in hemocytes of shrimp challenged by WSSV, immunocytochemical assays were performed following previous report [63]. Hemocytes were collected in 4% paraformaldehyde and anticoagulation mixtures (1:1) at different time points after WSSV challenge. The hemocytes were then washed three times with PBS and centrifuged at 800 × *g* for 6 min at 4 °C to remove the plasma. After re-suspending in PBS, the hemocytes were dropped onto poly-lysine coated glass slides and left to stand for 1 h. The slides were washed six times, and blocked with 3% bovine serum albumin (dissolved in PBS) for 30 min at 37 °C. Anti-*Mj*pIgR antibody was then added (1:100 diluted in 3% bovine serum albumin) and the cells were incubated over night at 4 °C. The hemocytes were washed with PBS six times, incubated with goat anti-rabbit antibody conjugated with ALEXA 488 (1:1000 diluted in PBS) for 2 h at 37 °C, washed with PBS again, and then stained with 4-6-diamidino-2-phenylindole (DAPI) for 10 min at room temperature. After washing six times, the slides were examined under a fluorescent microscope (Olympus BX51, Japan).

For the immunocytochemical assay of *Mj*clathrin, rabbit anti-clathrin heavy chain (Bioss, Beijing, China) was used (1:1000 diluted in 3% bovine serum albumin) as the primary antibody. The other steps were the same as those described above.

### Co-localization of fluorescent-labeled WSSV and *Mj*pIgR

To detect the interaction of WSSV particles with *Mj*pIgR, the purified WSSV particles were labeled with Dil (Beyotime, Shanghai, China) by incubation with Dil reagent (25 μg/ml) for 2 h at 37 °C and then centrifuged at 12000 × *g* for 20 min at 4 °C to remove the supernatant. The sediment was washed with PBS twice and resuspended in PBS. The Dil-labeled WSSV was injected into shrimps and hemocytes were collected at different times (0, 15, 30, and 60 min). The cells were subjected to immunocytochemical assays using anti-*Mj*pIgR antibodies to detect the colocalization of WSSV with *Mj*pIgR.

### Pull-down assay

Pull-down assays were performed to further explore the interaction between *Mj*pIgR and WSSV envelope proteins. The four main envelope proteins of WSSV (VP19, VP24, VP26, and VP28) were recombinantly expressed in *E*. *coli* using recombinant vector pET32A-VPs. Purified GST-tagged *Mj*pIgR (200 μg) was incubated with the four His-tagged envelope proteins (1:1), separately, for 5 h at 4 °C. After incubation with GST-bound resin (50 μl) for 45 min at 4 °C, the resin was washed with PBS five times. Elution buffer (10 mM reduced glutathione, 50 mM Tris-HCl, pH 8.0) was added to wash out the bound proteins. SDS-PAGE was conducted to analyze the proteins. His-pulldown was also performed. Purified His-tagged VPs was incubated with GST-tagged *Mj*pIgR, respectively. After incubation with His-bound resin for 45 min at 4 °C, the resin was washed with PBS five times. Elution buffer (0.5 M NaCl, 1 M imidazole, 20 mM Tris-HCl, pH 8.0) was used to wash out the bound protein.

To further confirm the interaction of *Mj*pIgR with VP24, the expression of truncating mutation of IG1 of *Mj*pIgR was performed. The sequence of *Mj*pIgR-ΔIG1 was amplified with primers *Mj*pIgR-ΔIG1-EX-F and *Mj*pIgR-ΔIG1-EX-R (Table 1) and cloned into pGEX-4T-1 vector for recombinant expression. The purified GST-tagged *Mj*pIgR-ΔIG1 was used for pulldown analysis.

### Enzyme-linked immunosorbent assay (ELISA)

Flat-bottomed 96-well microliter plates were coated with purified WSSV particles (50 μl) overnight at 4 °C, washed with TBST five times, and then blocked with 3% bovine serum albumin (dissolved in TBST) for 1 h at 4 °C. Different proteins were added to the plates at different concentrations. After incubation for 4 h at room temperature and washing five times, an anti-GST Tag Mouse monoclonal antibody (mAb; Abbkine, CA, USA) was added to the plates and incubated overnight at 4 °C. Horse anti-mouse antibody (Zsbio, Beijing, China) (1:2000 diluted in 3% bovine serum albumin) was added and incubated for 2 h at room temperature. After washing five times, 100 μl of the chromogenic reaction liquid (1 mg/ml p-nitro-phenyl phosphate, 10 mM diethanolamine, 0.5 mM MgCl_2_) was added to each well for 20 min at room temperature. The absorbance of each well was read using a Universal Microplate Reader ELX800 (Bio-Tek, USA) at 405 nm.

### Calmodulin antagonist assay

N-(4-Aminobutyl)-5-chloro-2-naphthalenesulfonamide hydrochloride W13 (W-13, Sigma-Aldrich, USA) was used as a calmodulin antagonist. Different concentrations of W-13 (10, 15, 30, 45, and 60 μM) were injected into shrimp, and WSSV particles were injected 2 h later. The WSSV expression levels were detected in the hemocytes and intestine at 24 h after WSSV infection.

### Chlorpromazine injection

To detect the endocytosis of WSSV, an inhibitor of clathrin-dependent endocytosis, chlorpromazine (CPZ, Sangon Biotech, Shanghai, China) was injected into shrimp at different concentrations (10, 15, 30, 45, and 60 μM) and WSSV was injected 2 h later. The amount of WSSV in the hemocytes and intestine was detected using qPCR. To analyze whether the *Mj*pIgR-induced WSSV endocytosis is clathrin-dependent, CPZ was injected into *Mj*pIgR-overexpressing shrimp infected with WSSV. WSSV replication was detected using qPCR and western blotting with an envelope protein of WSSV as the indicator.

### Flow cytometry

WSSV particles were labeled with Dil (red) for 2 h and then collected by centrifugation at 12000 × *g* for 20 min. The Dil-labeled WSSV particles were washed with PBS twice, and then suspended in PBS for shrimp injection. Hemocytes were collected at 1 h for overexpression or RNA interference, and detected using flow cytometry (ImageStreamX MarkII, USA).

### Oligomerization test

The *Mj*pIgR (extracellular domain) recombinant was used for native PAGE to detect the oligomerization in *vitro*, as described in previous articles [40]. A crosslinking assay was performed to detect oligomerization in *vivo*. Intestines from shrimp were ground into a homogenate in PBS, and Subric acid bis sodium salt (3-sulfo-N-hydroxysuccinimide ester, BS3; Sigma-Aldrich, USA) was added to a final concentration of 5 mM. After incubation for 2 h at room temperature, SDS-PAGE sample loading buffer was added for reaction termination. The reagent mixture was treated in a boiling water bath for 5 min followed by SDS-PAGE and western blotting.

## Supporting information

S1 Fig Architecture representation of Fasciclin proteins from *Drosophila melanogaster*, *M*. *japonicus*, and other insects predicted by SMART. *Apis cerana*, XP_016913112; *Drosophila melanogaster* Fasciclin I, AAF55346.2; Fasciclin II, AAF45925.2; Fasciclin III, NP_724107.1. *Musca domestica*, XP_005182792.
(TIF)

S2 Fig Domain architecture of pIgR analyzed by SMART (http://smart.embl-heidelberg.de/) in different species. *Homo sapiens*, AAI10495.1; *Danio rerio*, XM_021466408; *Poecilia latipinna*, XP_014912501; *Boleophthalmus pectinirostris*, XP_020786989; *Lepisosteus oculatus*, XP_015197895.
(TIF)

S3 Fig Phylogenetic analysis of pIgRs from *M*. *japonicus* and other species constructed by MEGA 5.0. (TIF)

S4 Fig Specificity analysis of *Mj*pIgR primers. The primers of *Mj*pIgR ORF were used for RT-PCR amplification with samples from *Litopenaeus vannamei and Procambarus clarkii*. No any band was detected in hemocytes and different organs of the two species.
(TIF)

S5 Fig Knockdown of *Mjβ-integrin* in shrimp inhibited WSSV replication and increased survival rate of the shrimp. **A**, The efficiency of *Mjβ-integrin* RNAi. **B**, The expression of WSSV *vp28* in *Mjβ-integrin* knockdown shrimp infected with WSSV. **C**, Survival rates of *Mjβ-integrin* knockdown and *dsGFP* injection shrimp after WSSV infection. Significant differences were analyzed using the software GraphPad Prism 5.0.
(TIF)

## References

[1] Sun E, He J, Zhuang X (2013) Live cell imaging of viral entry. Curr Opin Virol 3: 34–43. 10.1016/j.coviro.2013.01.005 23395264 PMC3587724

[2] Staring J, Raaben M, Brummelkamp TR (2018) Viral escape from endosomes and host detection at a glance. J Cell Sci 131. 10.1242/jcs.216259 30076240

[3] Seisenberger G, Ried MU, Endress T, Buning H, Hallek M, et al (2001) Real-time single-molecule imaging of the infection pathway of an adeno-associated virus. Science 294: 1929–1932. 10.1126/science.1064103 11729319

[4] Boulant S, Stanifer M, Lozach PY (2015) Dynamics of virus-receptor interactions in virus binding, signaling, and endocytosis. Viruses 7: 2794–2815. 10.3390/v7062747 26043381 PMC4488714

[5] Baranowski E, Ruiz-Jarabo CM, Domingo E (2001) Evolution of cell recognition by viruses. Science 292: 1102–1105. 11352064 10.1126/science.1058613

[6] Stehle T, Casasnovas JM (2009) Specificity switching in virus-receptor complexes. Curr Opin Struct Biol 19: 181–188. 10.1016/j.sbi.2009.02.013 19342221 PMC7126087

[7] Bhella D (2015) The role of cellular adhesion molecules in virus attachment and entry. Philos Trans R Soc Lond B Biol Sci 370: 20140035 10.1098/rstb.2014.0035 25533093 PMC4275905

[8] Barth H, Schafer C, Adah MI, Zhang F, Linhardt RJ, et al (2003) Cellular binding of hepatitis C virus envelope glycoprotein E2 requires cell surface heparan sulfate. J Biol Chem 278: 41003–41012. 10.1074/jbc.M302267200 12867431

[9] Agnello V, Abel G, Elfahal M, Knight GB, Zhang QX (1999) Hepatitis C virus and other flaviviridae viruses enter cells via low density lipoprotein receptor. Proc Natl Acad Sci U S A 96: 12766–12771. 10535997 10.1073/pnas.96.22.12766 PMC23090

[10] Martin DN, Uprichard SL (2013) Identification of transferrin receptor 1 as a hepatitis C virus entry factor. Proc Natl Acad Sci U S A 110: 10777–10782. 10.1073/pnas.1301764110 23754414 PMC3696786

[11] Scarselli E, Ansuini H, Cerino R, Roccasecca RM, Acali S, et al (2002) The human scavenger receptor class B type I is a novel candidate receptor for the hepatitis C virus. EMBO J 21: 5017–5025. 10.1093/emboj/cdf529 12356718 PMC129051

[12] Ploss A, Evans MJ, Gaysinskaya VA, Panis M, You H, et al (2009) Human occludin is a hepatitis C virus entry factor required for infection of mouse cells. Nature 457: 882–886. 10.1038/nature07684 19182773 PMC2762424

[13] Samanta D, Almo SC (2015) Nectin family of cell-adhesion molecules: structural and molecular aspects of function and specificity. Cell Mol Life Sci 72: 645–658. 10.1007/s00018-014-1763-4 25326769 PMC11113404

[14] Williams AF, Barclay AN (1988) The immunoglobulin superfamily—domains for cell surface recognition. Annu Rev Immunol 6: 381–405. 10.1146/annurev.iy.06.040188.002121 3289571

[15] Dermody TS, Kirchner E, Guglielmi KM, Stehle T (2009) Immunoglobulin superfamily virus receptors and the evolution of adaptive immunity. PLoS Pathog 5: e1000481 10.1371/journal.ppat.1000481 19956667 PMC2777377

[16] Asano M, Komiyama K (2011) Polymeric immunoglobulin receptor. J Oral Sci 53: 147–156. 21712618 10.2334/josnusd.53.147

[17] Kaetzel CS (2005) The polymeric immunoglobulin receptor: bridging innate and adaptive immune responses at mucosal surfaces. Immunol Rev 206: 83–99. 10.1111/j.0105-2896.2005.00278.x 16048543

[18] Kaetzel CS, Robinson JK, Chintalacharuvu KR, Vaerman JP, Lamm ME (1991) The polymeric immunoglobulin receptor (secretory component) mediates transport of immune complexes across epithelial cells: a local defense function for IgA. Proc Natl Acad Sci U S A 88: 8796–8800. 1924341 10.1073/pnas.88.19.8796 PMC52597

[19] Hamuro K, Suetake H, Saha NR, Kikuchi K, Suzuki Y (2007) A teleost polymeric Ig receptor exhibiting two Ig-like domains transports tetrameric IgM into the skin. J Immunol 178: 5682–5689. 17442951 10.4049/jimmunol.178.9.5682

[20] Robinson JK, Blanchard TG, Levine AD, Emancipator SN, Lamm ME (2001) A mucosal IgA-mediated excretory immune system in vivo. J Immunol 166: 3688–3692. 11238608 10.4049/jimmunol.166.6.3688

[21] Phalipon A, Cardona A, Kraehenbuhl JP, Edelman L, Sansonetti PJ, et al (2002) Secretory component: a new role in secretory IgA-mediated immune exclusion in vivo. Immunity 17: 107–115. 12150896 10.1016/s1074-7613(02)00341-2

[22] Zhang JR, Mostov KE, Lamm ME, Nanno M, Shimida S, et al (2000) The polymeric immunoglobulin receptor translocates pneumococci across human nasopharyngeal epithelial cells. Cell 102: 827–837. 11030626 10.1016/s0092-8674(00)00071-4

[23] Kaetzel CS (2001) Polymeric Ig receptor: defender of the fort or Trojan horse? Curr Biol 11: R35–38. 11166195 10.1016/s0960-9822(00)00041-5

[24] Brock SC, McGraw PA, Wright PF, Crowe JE Jr. (2002) The human polymeric immunoglobulin receptor facilitates invasion of epithelial cells by Streptococcus pneumoniae in a strain-specific and cell type-specific manner. Infect Immun 70: 5091–5095. 10.1128/IAI.70.9.5091-5095.2002 12183558 PMC128237

[25] Dalgleish AG, Beverley PC, Clapham PR, Crawford DH, Greaves MF, et al (1984) The CD4 (T4) antigen is an essential component of the receptor for the AIDS retrovirus. Nature 312: 763–767. 6096719 10.1038/312763a0

[26] Pandey D, Podder A, Pandit M, Latha N (2016) CD4-gp120 interaction interface—a gateway for HIV-1 infection in human: molecular network, modeling and docking studies. J Biomol Struct Dyn: 1–14. 10.1080/07391102.2016.1227722 27545652

[27] Ryser HJ, Fluckiger R (2005) Progress in targeting HIV-1 entry. Drug Discov Today 10: 1085–1094. 10.1016/S1359-6446(05)03550-6 16182193

[28] Berger EA, Murphy PM, Farber JM (1999) Chemokine receptors as HIV-1 coreceptors: roles in viral entry, tropism, and disease. Annu Rev Immunol 17: 657–700. 10.1146/annurev.immunol.17.1.657 10358771

[29] Barbouche R, Miquelis R, Jones IM, Fenouillet E (2003) Protein-disulfide isomerase-mediated reduction of two disulfide bonds of HIV envelope glycoprotein 120 occurs post-CXCR4 binding and is required for fusion. J Biol Chem 278: 3131–3136. 10.1074/jbc.M205467200 12218052

[30] Pillay S, Meyer NL, Puschnik AS, Davulcu O, Diep J, et al (2016) An essential receptor for adeno-associated virus infection. Nature 530: 108–112. 10.1038/nature16465 26814968 PMC4962915

[31] Peng SE, Lo CF, Lin SC, Chen LL, Chang YS, et al (2001) Performance of WSSV-infected and WSSV-negative Penaeus monodon postlarvae in culture ponds. Dis Aquat Organ 46: 165–172. 10.3354/dao046165 11710550

[32] Sritunyalucksana K, Wannapapho W, Lo CF, Flegel TW (2006) PmRab7 is a VP28-binding protein involved in white spot syndrome virus infection in shrimp. J Virol 80: 10734–10742. 10.1128/JVI.00349-06 17041224 PMC1641754

[33] Chen LL, Lu LC, Wu WJ, Lo CF, Huang WP (2007) White spot syndrome virus envelope protein VP53A interacts with Penaeus monodon chitin-binding protein (PmCBP). Dis Aquat Organ 74: 171–178. 10.3354/dao074171 17465302

[34] Chen KY, Hsu TC, Huang PY, Kang ST, Lo CF, et al (2009) Penaeus monodon chitin-binding protein (PmCBP) is involved in white spot syndrome virus (WSSV) infection. Fish Shellfish Immunol 27: 460–465. 10.1016/j.fsi.2009.06.018 19576286

[35] Li DF, Zhang MC, Yang HJ, Zhu YB, Xu X (2007) Beta-integrin mediates WSSV infection. Virology 368: 122–132. 10.1016/j.virol.2007.06.027 17655902

[36] Huang HT, Leu JH, Huang PY, Chen LL (2012) A putative cell surface receptor for white spot syndrome virus is a member of a transporter superfamily. PLoS One 7: e33216 10.1371/journal.pone.0033216 22427993 PMC3302809

[37] Liu WJ, Li YC, Kou GH, Lo CF (2016) Laminin Receptor in Shrimp Is a Cellular Attachment Receptor for White Spot Syndrome Virus. PLoS One 11: e0156375 10.1371/journal.pone.0156375 27257954 PMC4892510

[38] Wang XW, Xu YH, Xu JD, Zhao XF, Wang JX (2014) Collaboration between a soluble C-type lectin and calreticulin facilitates white spot syndrome virus infection in shrimp. J Immunol 193: 2106–2117. 10.4049/jimmunol.1400552 25070855

[39] Liang Y, Cheng JJ, Yang B, Huang J (2010) The role of F1 ATP synthase beta subunit in WSSV infection in the shrimp, Litopenaeus vannamei. Virol J 7: 144 10.1186/1743-422X-7-144 20591132 PMC2906456

[40] Yang MC, Shi XZ, Yang HT, Sun JJ, Xu L, et al (2016) Scavenger Receptor C Mediates Phagocytosis of White Spot Syndrome Virus and Restricts Virus Proliferation in Shrimp. PLoS Pathog 12: e1006127 10.1371/journal.ppat.1006127 28027319 PMC5222524

[41] Mercer J, Schelhaas M, Helenius A (2010) Virus entry by endocytosis. Annu Rev Biochem 79: 803–833. 10.1146/annurev-biochem-060208-104626 20196649

[42] Merisko EM, Welch JK, Chen TY, Chen M (1988) Alpha-actinin and calmodulin interact with distinct sites on the arms of the clathrin trimer. J Biol Chem 263: 15705–15712. 3170607

[43] Chapin SJ, Enrich C, Aroeti B, Havel RJ, Mostov KE (1996) Calmodulin binds to the basolateral targeting signal of the polymeric immunoglobulin receptor. J Biol Chem 271: 1336–1342. 8576121 10.1074/jbc.271.3.1336

[44] Conner SD, Schmid SL (2003) Regulated portals of entry into the cell. Nature 422: 37–44. 10.1038/nature01451 12621426

[45] Robinson MS (2004) Adaptable adaptors for coated vesicles. Trends Cell Biol 14: 167–174. 10.1016/j.tcb.2004.02.002 15066634

[46] Motley A, Bright NA, Seaman MN, Robinson MS (2003) Clathrin-mediated endocytosis in AP-2-depleted cells. J Cell Biol 162: 909–918. 10.1083/jcb.200305145 12952941 PMC2172830

[47] Wai Wong C, Dye DE, Coombe DR (2012) The role of immunoglobulin superfamily cell adhesion molecules in cancer metastasis. Int J Cell Biol 2012: 340296 10.1155/2012/340296 22272201 PMC3261479

[48] Juliano RL (2002) Signal transduction by cell adhesion receptors and the cytoskeleton: functions of integrins, cadherins, selectins, and immunoglobulin-superfamily members. Annu Rev Pharmacol Toxicol 42: 283–323. 10.1146/annurev.pharmtox.42.090401.151133 11807174

[49] Barclay AN (2003) Membrane proteins with immunoglobulin-like domains—a master superfamily of interaction molecules. Semin Immunol 15: 215–223. 14690046 10.1016/s1044-5323(03)00047-2

[50] Rogers GN, Paulson JC (1983) Receptor determinants of human and animal influenza virus isolates: differences in receptor specificity of the H3 hemagglutinin based on species of origin. Virology 127: 361–373. 6868370 10.1016/0042-6822(83)90150-2

[51] Bergelson JM, Shepley MP, Chan BM, Hemler ME, Finberg RW (1992) Identification of the integrin VLA-2 as a receptor for echovirus 1. Science 255: 1718–1720. 1553561 10.1126/science.1553561

[52] Pulli T, Koivunen E, Hyypia T (1997) Cell-surface interactions of echovirus 22. J Biol Chem 272: 21176–21180. 9261123 10.1074/jbc.272.34.21176

[53] Pal K, Kaetzel CS, Brundage K, Cunningham CA, Cuff CF (2005) Regulation of polymeric immunoglobulin receptor expression by reovirus. J Gen Virol 86: 2347–2357. 10.1099/vir.0.80690-0 16033983

[54] Mercer J, Greber UF (2013) Virus interactions with endocytic pathways in macrophages and dendritic cells. Trends Microbiol 21: 380–388. 10.1016/j.tim.2013.06.001 23830563

[55] Verbruggen B, Bickley LK, van Aerle R, Bateman KS, Stentiford GD, et al (2016) Molecular Mechanisms of White Spot Syndrome Virus Infection and Perspectives on Treatments. Viruses 8. 10.3390/v8010023 PMC4728583 26797629

[56] Escobedo-Bonilla CM, Alday-Sanz V, Wille M, Sorgeloos P, Pensaert MB, et al (2008) A review on the morphology, molecular characterization, morphogenesis and pathogenesis of white spot syndrome virus. J Fish Dis 31: 1–18. 10.1111/j.1365-2761.2007.00877.x 18086030

[57] Merisko EM (1985) Evidence for the interaction of alpha-actinin and calmodulin with the clathrin heavy chain. Eur J Cell Biol 39: 167–172. 2867905

[58] Pley UM, Hill BL, Alibert C, Brodsky FM, Parham P (1995) The interaction of calmodulin with clathrin-coated vesicles, triskelions, and light chains. Localization of a binding site. J Biol Chem 270: 2395–2402. 7836475 10.1074/jbc.270.5.2395

[59] Wang S, Zhao XF, Wang JX (2009) Molecular cloning and characterization of the translationally controlled tumor protein from Fenneropenaeus chinensis. Mol Biol Rep 36: 1683–1693. 10.1007/s11033-008-9369-2 18853281

[60] Du XJ, Zhao XF, Wang JX (2007) Molecular cloning and characterization of a lipopolysaccharide and beta-1,3-glucan binding protein from fleshy prawn (Fenneropenaeus chinensis). Mol Immunol 44: 1085–1094. 10.1016/j.molimm.2006.07.288 16930711

[61] Livak KJ, Schmittgen TD (2001) Analysis of relative gene expression data using real-time quantitative PCR and the 2(-Delta Delta C(T)) Method. Methods 25: 402–408. 10.1006/meth.2001.1262 11846609

[62] Wang XW, Gao J, Xu YH, Xu JD, Fan ZX, et al (2017) Novel Pattern Recognition Receptor Protects Shrimp by Preventing Bacterial Colonization and Promoting Phagocytosis. J Immunol 198: 3045–3057. 10.4049/jimmunol.1602002 28258197

[63] Sun JJ, Lan JF, Zhao XF, Vasta GR, Wang JX (2017) Binding of a C-type lectin's coiled-coil domain to the Domeless receptor directly activates the JAK/STAT pathway in the shrimp immune response to bacterial infection. PLoS Pathog 13: e1006626 10.1371/journal.ppat.1006626 28931061 PMC5645147

