# Supplementary figures and images for: The polymeric immunoglobulin receptor-like protein from Marsupenaeus japonicus is a receptor for white spot syndrome virus infection
Source: PLoS Pathog. 2019 Feb 6;15(2):e1007558. doi: 10.1371/journal.ppat.1007558 (PMC6380602; doi:10.1371/journal.ppat.1007558)

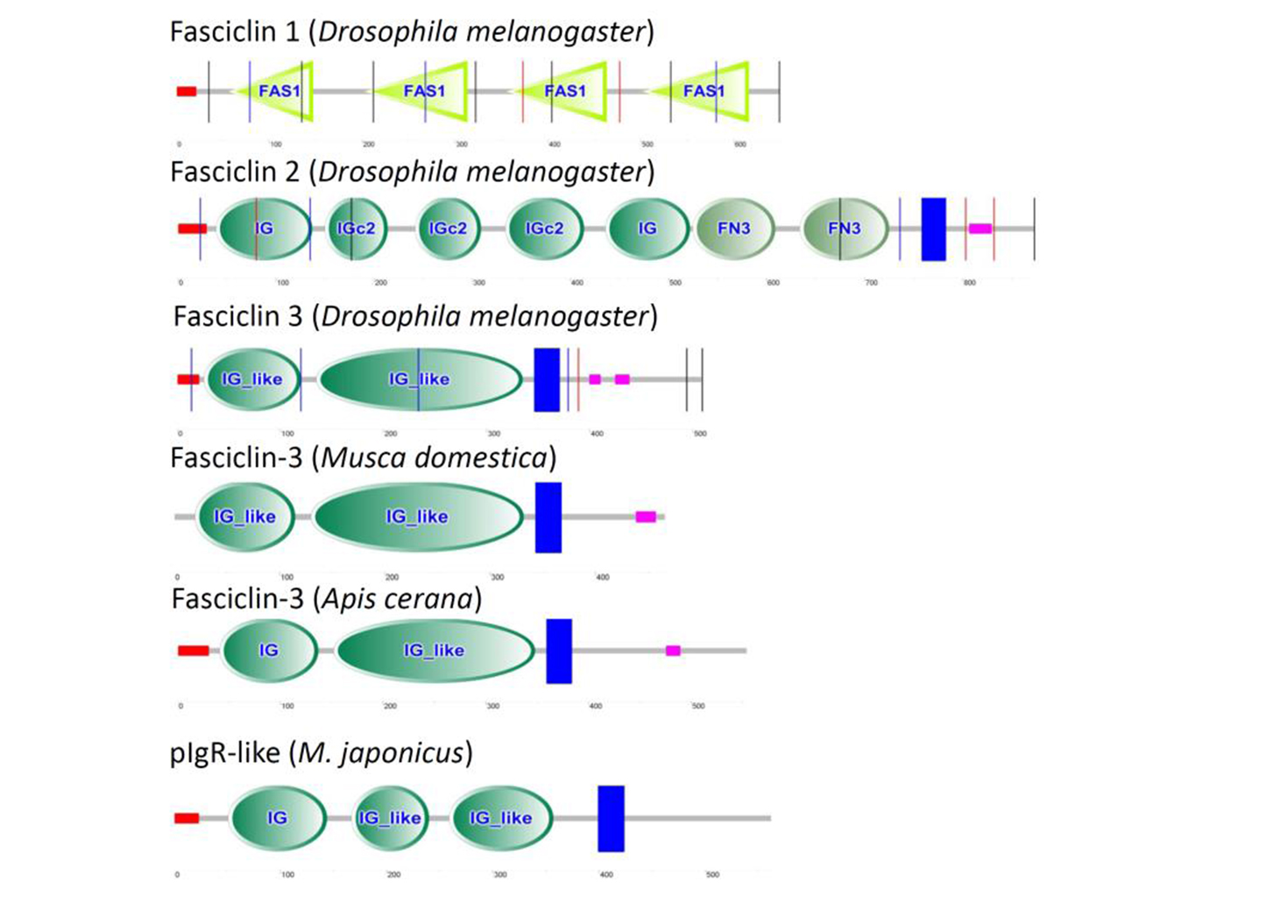

Supplement: S1 Fig — Apis cerana, XP_016913112; Drosophila melanogaster Fasciclin I, AAF55346.2; Fasciclin II, AAF45925.2; Fasciclin III, NP_724107.1. Musca domestica, XP_005182792. (TIF) [file ppat.1007558.s001.tif]

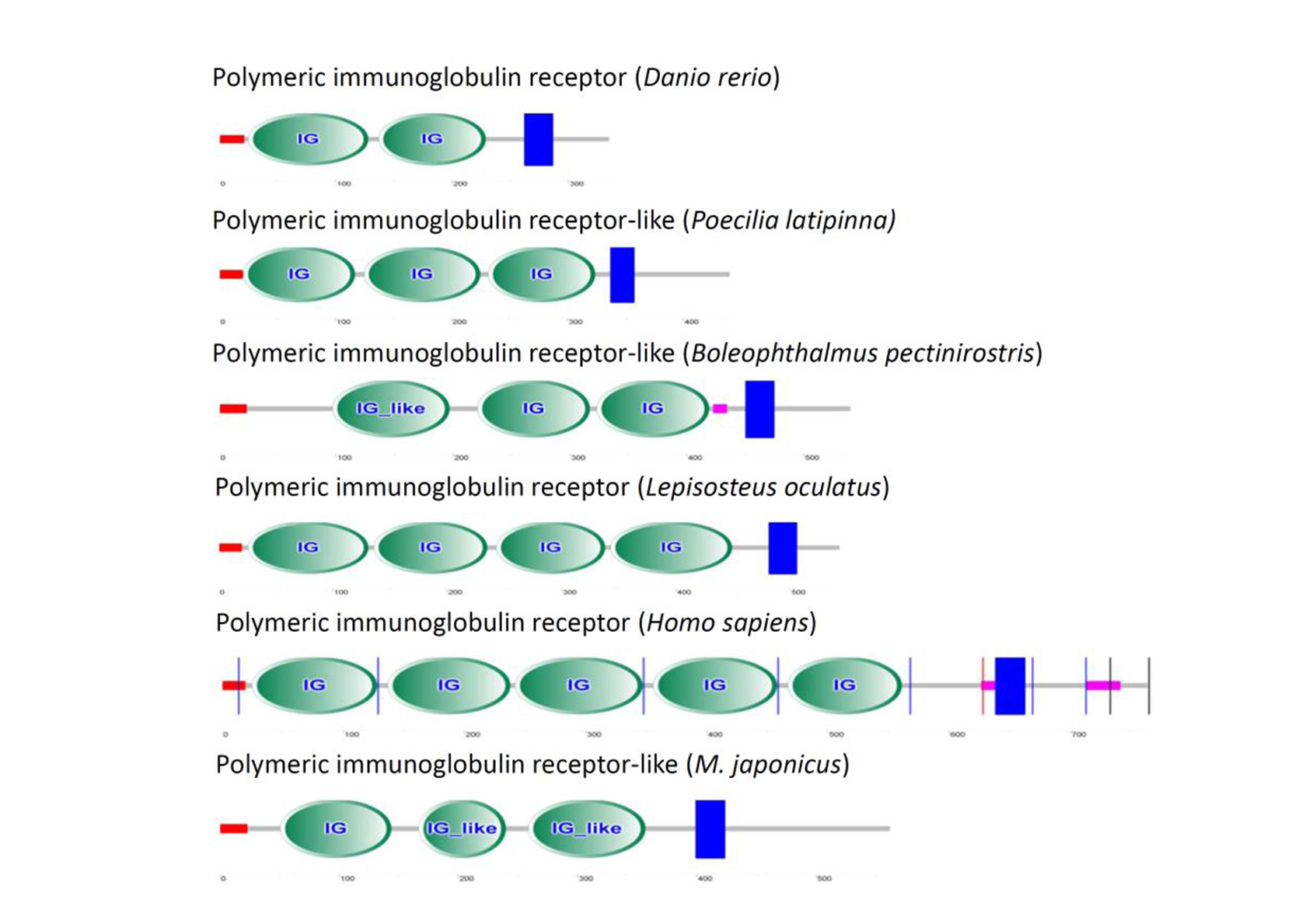

Supplement: S2 Fig — Homo sapiens, AAI10495.1; Danio rerio, XM_021466408; Poecilia latipinna, XP_014912501; Boleophthalmus pectinirostris, XP_020786989; Lepisosteus oculatus, XP_015197895. (TIF) [file ppat.1007558.s002.tif]

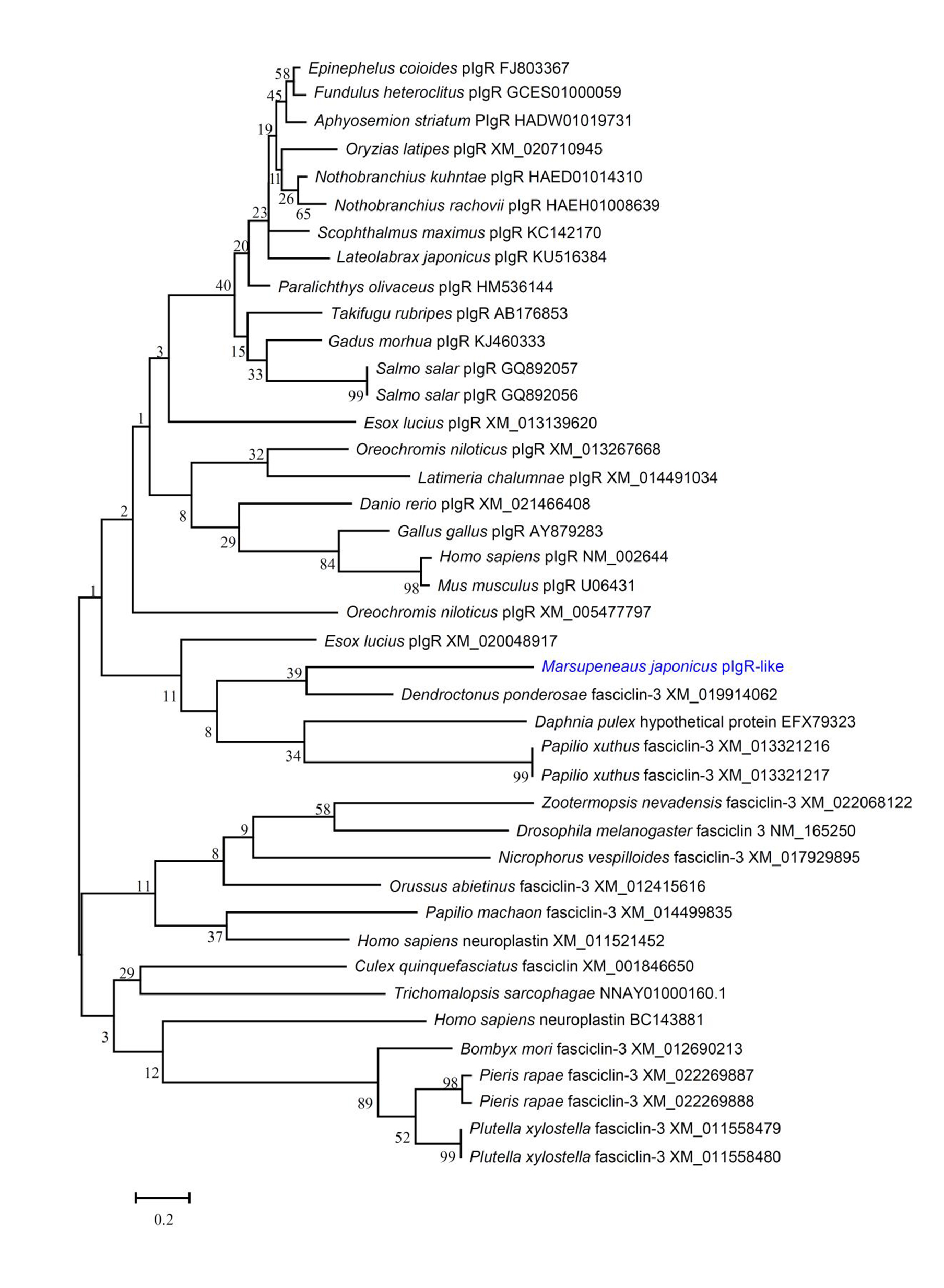

Supplement: S3 Fig — (TIF) [file ppat.1007558.s003.tif]

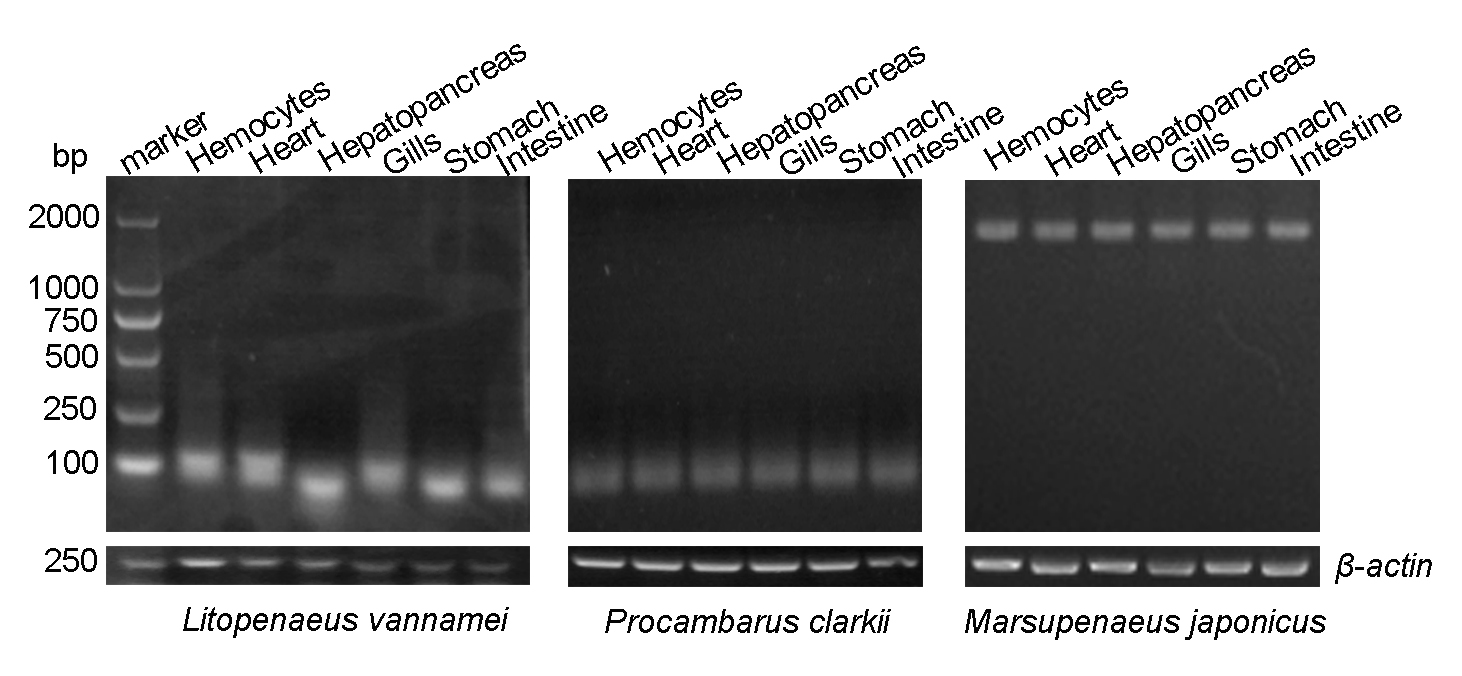

Supplement: S4 Fig — The primers of MjpIgR ORF were used for RT-PCR amplification with samples from Litopenaeus vannamei and Procambarus clarkii. No any band was detected in hemocytes and different organs of the two species. (TIF) [file ppat.1007558.s004.tif]

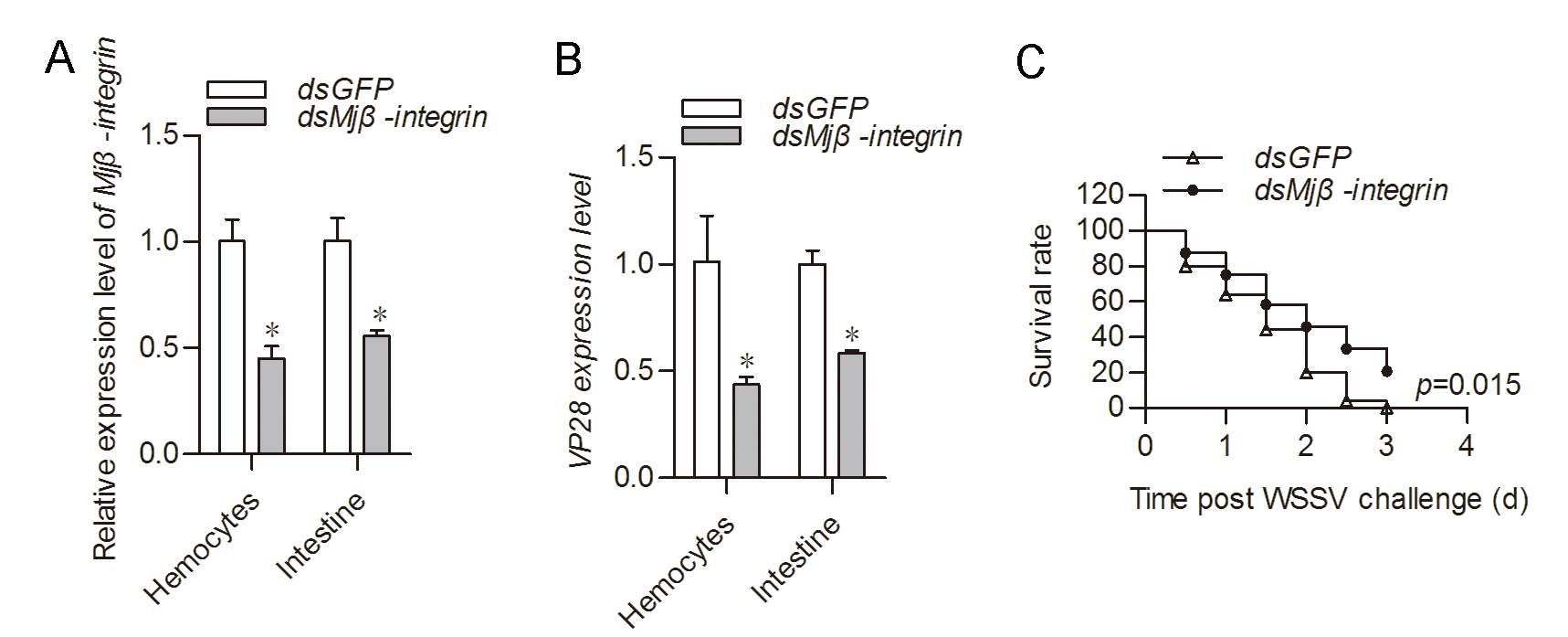

Supplement: S5 Fig — A, The efficiency of Mjβ-integrin RNAi. B, The expression of WSSV vp28 in Mjβ-integrin knockdown shrimp infected with WSSV. C, Survival rates of Mjβ-integrin knockdown and dsGFP injection shrimp after WSSV infection. Significant differences were analyzed using the software GraphPad Prism 5.0. (TIF) [file ppat.1007558.s005.tif]
